# NODAL/TGFβ signalling mediates the self-sustained stemness induced by *PIK3CA^H1047R^* homozygosity in pluripotent stem cells

**DOI:** 10.1242/dmm.048298

**Published:** 2021-03-11

**Authors:** Ralitsa R. Madsen, James Longden, Rachel G. Knox, Xavier Robin, Franziska Völlmy, Kenneth G. Macleod, Larissa S. Moniz, Neil O. Carragher, Rune Linding, Bart Vanhaesebroeck, Robert K. Semple

**Affiliations:** 1 Centre for Cardiovascular Science, Queen's Medical Research Institute, University of Edinburgh, Edinburgh EH16 4TJ, UK; 2 Metabolic Research Laboratories, Wellcome Trust-MRC Institute of Metabolic Science, University of Cambridge, Cambridge CB2 0QQ, UK; 3 The National Institute for Health Research Cambridge Biomedical Research Centre, Cambridge CB2 0QQ, UK; 4 Biotech Research and Innovation Centre, University of Copenhagen, DK-2200 Copenhagen, Denmark; 5 Theoretical Biophysics, Institute of Biology, Humboldt-Universität zu Berlin, 10115 Berlin, Germany; 6 Edinburgh Cancer Research UK Centre, Institute of Genetics and Molecular Medicine, University of Edinburgh, Western General Hospital, Crewe Road South, Edinburgh EH4 2XR, UK; 7 University College London Cancer Institute, Paul O'Gorman Building, University College London, London WC1E 6BT, UK

**Keywords:** PI3K, *PIK3CA*, Stemness, Pluripotent stem cells

## Abstract

Activating *PIK3CA* mutations are known ‘drivers’ of human cancer and developmental overgrowth syndromes. We recently demonstrated that the ‘hotspot’ *PIK3CA^H1047R^* variant exerts unexpected allele dose-dependent effects on stemness in human pluripotent stem cells (hPSCs). In this study, we combine high-depth transcriptomics, total proteomics and reverse-phase protein arrays to reveal potentially disease-related alterations in heterozygous cells, and to assess the contribution of activated TGFβ signalling to the stemness phenotype of homozygous *PIK3CA^H1047R^* cells. We demonstrate signalling rewiring as a function of oncogenic PI3K signalling strength, and provide experimental evidence that self-sustained stemness is causally related to enhanced autocrine NODAL/TGFβ signalling. A significant transcriptomic signature of TGFβ pathway activation in heterozygous *PIK3CA^H1047R^* was observed but was modest and was not associated with the stemness phenotype seen in homozygous mutants. Notably, the stemness gene expression in homozygous *PIK3CA^H1047R^* hPSCs was reversed by pharmacological inhibition of NODAL/TGFβ signalling, but not by pharmacological PI3Kα pathway inhibition. Altogether, this provides the first in-depth analysis of PI3K signalling in hPSCs and directly links strong PI3K activation to developmental NODAL/TGFβ signalling. This work illustrates the importance of allele dosage and expression when artificial systems are used to model human genetic disease caused by activating *PIK3CA* mutations.

This article has an associated First Person interview with the first author of the paper.

## Abstract (precis)

**Summary:** Transcriptomic and proteomic analyses of iPSCs with allele-dose dependent expression of the *PIK3CA^H1047R^* oncogene confirm network rewiring in homozygous mutants, with self-sustained stemness likely driven by constitutive TGFβ/NODAL pathway activation.

## INTRODUCTION

Class IA phosphoinositide 3-kinases (PI3Ks) are evolutionarily conserved enzymes that catalyse formation of the membrane-bound second messenger phosphatidylinositol-3,4,5-trisphosphate (PIP_3_). PI3Ks are activated downstream of receptor tyrosine kinases, with the ensuing increase in PIP_3_ and its derivative PI(3,4)P_2_ triggering a widespread signalling network, best known for the activation of the serine/threonine kinases AKT and mTORC1. PI3K activation promotes cell survival, glucose uptake, anabolic metabolism, cell proliferation and cell migration (Fruman et al., 2017). Among the class IA PI3K isoforms (PI3Kα, PI3Kβ, PI3Kδ), the ubiquitously expressed PI3Kα (encoded by the *PIK3CA* gene in humans) is the main regulator of organismal growth, development and survival (Bilanges et al., 2019).

Activating mutations in *PIK3CA* are among the most common somatic point mutations in cancer, together with inactivation or loss of the tumour suppressor *PTEN* (a negative regulator of PI3K) (Chang et al., 2015; Sanchez-Vega et al., 2018; Campbell et al., 2020). The same mutations in *PIK3CA*, when acquired postzygotically during development, also cause a range of largely benign overgrowth disorders (Madsen et al., 2018), for which the term *PIK3CA*-related overgrowth spectrum (PROS) has been coined. Motivated by the need to understand the role of PI3K signalling in a human developmental context, we previously generated an allelic series of human induced pluripotent stem cells (iPSCs) with heterozygous or homozygous expression of the activating mutation *PIK3CA^H1047R^*, the most commonly observed *PIK3CA* mutation in both cancer and PROS (Madsen et al., 2019). Despite the severe developmental disorders caused by heterozygosity for *PIK3CA^H1047R^* in humans *in vivo*, we found little discernible effect on germ layer specification from heterozygous iPSCs. In sharp contrast, homozygosity for *PIK3CA^H1047R^* led to self-sustained stemness and resistance to spontaneous differentiation *in vitro* and *in vivo* (Madsen et al., 2019). This suggested a previously unappreciated quantitative relationship between the strength of PI3K signalling and the gene regulatory network (GRN) in pluripotent stem cells.

The core pluripotency GRN features a feedforward autoregulatory circuit comprising three transcription factors, namely SRY box 2 (SOX2), Octamer-binding transcription factor 3/4 (OCT3/4; encoded by *POU5F1*), and the homeobox transcription factor NANOG (Boyer et al., 2005; Loh et al., 2006; Li and Belmonte, 2017). SOX2 helps sustain OCT3/4 expression, which is required for the establishment and maintenance of the pluripotent state (Nichols et al., 1998). However, even modest overexpression of OCT3/4 destabilises the pluripotency network and triggers differentiation (Niwa et al., 2000; Radzisheuskaya et al., 2013). In contrast, NANOG, although dispensable for the maintenance of pluripotency (Chambers et al., 2007), stabilises the pluripotency GRN. Overexpression of *NANOG* by as little as 1.5-fold leads to sustained self-renewal (or ‘stemness’) of murine and human PSCs (hPSCs) (Chambers et al., 2003; Mitsui et al., 2003; Darr, 2006; Ivanova et al., 2006). In hPSCs, NANOG expression is activated by the transcription factors SMAD2/3 (Xu et al., 2008), which in turn are activated by receptors binding TGFβ, Activin or NODAL (Pauklin and Vallier, 2015). Overexpression of NODAL thus results in self-sustained stemness of hPSCs even in differentiation-promoting conditions (Vallier et al., 2004, 2005).

Given the unexpected and surprisingly mild phenotype caused by heterozygous *PIK3CA^H1047R^* expression in iPSCs, we reasoned that more sensitive assays would allow us to discern small but disease-relevant alterations in these cells. Thus, in this study, we first applied high-depth transcriptomics and proteomics to seek evidence of disease-related phenotypes in heterozygous cells, and to investigate how high-dose PI3K signalling leads to self-sustained stemness in homozygous *PIK3CA^H1047R^* iPSCs. We demonstrate that heterozygous cells do exhibit significant transcriptomic changes, although these are a weak echo of the widespread changes seen in homozygous cells. The mild transcriptional consequences of heterozygous expression of disease-relevant *PIK3CA* mutations were also validated in additional model systems and contrast with previous findings of major transcriptional rewiring in immortalised non-transformed breast epithelial cells (Hart et al., 2015; Kiselev et al., 2015). We demonstrate that the stemness phenotype of *PIK3CA^H1047R/H1047R^* iPSCs is maintained by self-sustained NODAL/TGFβ signalling, in line with increased *PIK3CA*-mediated *NODAL* expression, and that it is not reversible by PI3Kα-specific inhibition. This work provides in-depth characterisation of the near-binary PI3K signalling effects seen in hPSCs, and provides evidence for PI3Kα-induced NODAL/TGFβ signalling as the mechanism for self-sustained stemness in homozygous *PIK3CA^H1047R^* iPSCs. We discuss the implications of our findings for understanding and modelling developmental disorders and cancers driven by genetic PI3K activation.

## RESULTS

### A sharp PI3K activity threshold determines gene expression changes in *PIK3CA^H1047R^* iPSCs

We previously generated isogenic human iPSCs with heterozygous or homozygous knock-in of the ‘hotspot’ *PIK3CA^H1047R^* mutation. Surprisingly, heterozygous cells showed few phenotypic changes and differentially expressed protein-coding transcripts. In contrast, homozygous *PIK3CA^H1047R/H1047R^* cells exhibited marked morphological changes and altered gene expression, with strong enrichment for cancer-associated pathways (Madsen et al., 2019).

**Fig. 1.**
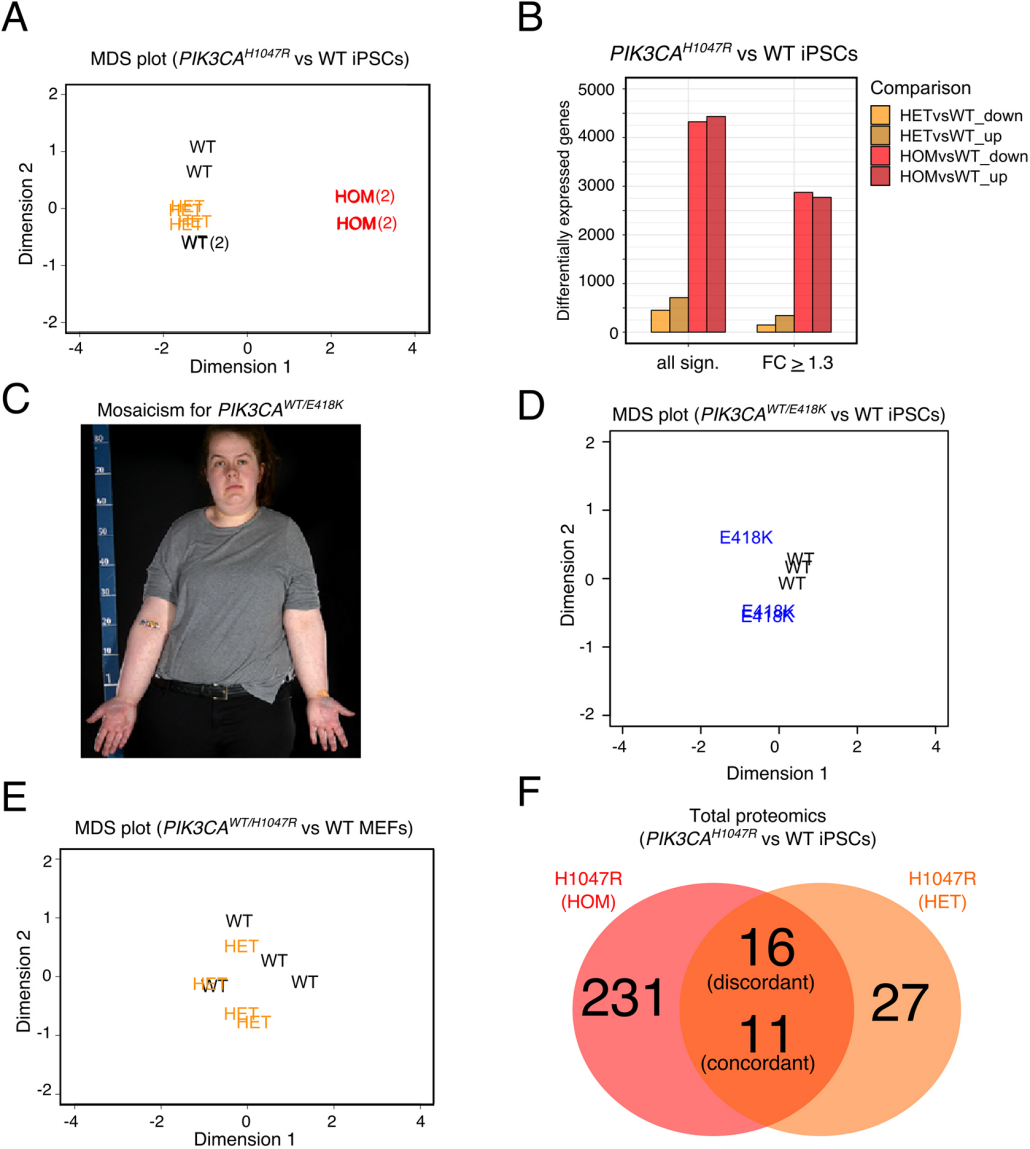
**Transcriptomic and proteomic analyses of human and mouse cell lines with endogenous expression of oncogenic *PIK3CA*.** (A) MDS plot of the transcriptomes of wild-type (WT), *PIK3CA^WT/H1047R^* (HET) and *PIK3CA^H1047R/H1047R^* (HOM) human iPSCs. The numbers in brackets indicate the presence of two closely overlapping samples. (B) The number of differentially expressed genes in iPSCs heterozygous or homozygous for *PIK3CA^H1047R^* before and after application of an absolute fold-change cutoff of ≥1.3 [false discovery rate (FDR), ≤0.05, Benjamini-Hochberg]. The data are based on four iPSC cultures from a minimum of two clones per genotype. See also Fig. S1. (C) Woman with asymmetric overgrowth caused by mosaicism for cells with heterozygous expression of *PIK3CA^E418K^*. Skin biopsies obtained from unaffected and affected tissues were used to obtain otherwise isogenic dermal fibroblasts for subsequent reprogramming into iPSCs. This image was reproduced from Parker et al., (2019). (D) MDS plot of the transcriptomes of wild-type (WT) and *PIK3CA^WT/E418K^* iPSCs (based on three independent mutant clones and three wild-type cultures from two independent clones). (E) MDS plot of the transcriptomes of wild-type (WT) and *PIK3CA^WT/H1047R^* (HET) MEFs following 48 h of mutant induction (*N*=4 independent clones per genotype). (F) Venn diagram showing the number of differentially expressed proteins in *PIK3CA^H1047R/H1047R^* (HOM) and *PIK3CA^WT/H1047R^* (HET) iPSCs relative to wild-type controls, profiled by label-free total proteomics on three clones per genotype. An absolute fold-change and *z*-score ≥1.2 were used to classify proteins as differentially expressed. The number of discordant and concordant changes in the expression of total proteins detected in both comparisons are indicated. See also Fig. S2.

To substantiate the apparent PI3K activity threshold manifest in *PIK3CA^H1047R^*-driven gene expression changes, and to look for further disease-related changes in heterozygous cells, we undertook RNA sequencing at substantially greater depth, also increasing the sample size to four and including previously unstudied iPSC clones for the wild-type and homozygous *PIK3CA* genotype. All clones were obtained from CRISPR/Cas9 editing of the same parental iPSC line (WTC11), which has been extensively validated and used for genetic engineering by the Allen Cell Collection (http://www.allencell.org/). As before, *PIK3CA^H1047R^* homozygous mutant cells clearly separated from heterozygous and wild-type cells, which overlapped on multidimensional scaling (MDS) (Fig. 1A), but we now detected a reduction in the levels of 451 transcripts and an increase in the levels of 710 transcripts in *PIK3CA^WT/H1047R^* iPSCs (Fig. 1B). This dropped to 149 and 343 transcripts, respectively, after applying a fold-change cutoff of 1.3 (Fig. 1B; Table S5), indicative of the small magnitude of many expression changes in heterozygous mutants (Fig. S1A). Use of the same cutoff of 1.3, in sharp distinction, yielded 2873 and 2771 transcripts of decreased or increased abundance, respectively, in homozygous iPSC mutants (Fig. 1B; Table S6). Not only was the number of gene expression changes higher by an order of magnitude in homozygous cells, but many expression changes were large compared to wild-type controls (Fig. S1A). The magnitudes of gene expression changes in *PIK3CA^H1047R/H1047R^* cells correlated strongly with our previous findings (Spearman's ρ=0.74, *P*<2e-16) (Fig. S1B), whereas correlation was low (Spearman's ρ=0.1, *P*<2e-16) for *PIK3CA^WT/H1047R^* iPSCs (Fig. S1C), as expected given the smaller number and lower magnitude of observed gene expression changes in heterozygous cells, and the lower depth of previous transcriptomic studies.

Given previous reports that *PIK3CA^H1047R^* heterozygosity in breast epithelial cells extensively remodels gene expression (Hart et al., 2015; Kiselev et al., 2015), we undertook further transcriptional profiling in two unrelated cellular models of genetic *PIK3CA* activation. First, we examined iPSCs derived from a woman with clinically obvious but mild PROS due to mosaicism for *PIK3CA^E418K^* (Fig. 1C) (Parker et al., 2019)*.* Heterozygous iPSCs were compared to wild-type lines established simultaneously from dermal fibroblasts from the same skin biopsy, which is possible due to genetic mosaicism of the sampled skin. Like *PIK3CA^WT/H1047R^* iPSCs, *PIK3CA^WT/E418K^* iPSCs closely clustered with isogenic wild-type controls on MDS plotting (Fig. 1D), with only 30 differentially expressed genes (Table S7). We also studied previously reported *Pik3ca^WT/H1047R^* mouse embryonic fibroblasts (MEFs) 48 h after *Cre*-mediated *Pik3ca^H1047R^* induction (Moniz et al., 2017). Wild-type and *Pik3ca^WT/H1047R^* MEFs were superimposable on an MDS plot (Fig. 1E), with only 192 downregulated and 77 upregulated genes (Table S8). Our findings suggest that there are bona fide transcriptional changes induced by heterozygosity for *PIK3CA^H1047R^*, but these are dramatically smaller in number and magnitude than changes induced by homozygosity for *PIK3CA^H1047R^*.

To assess whether transcriptional changes observed in iPSCs were mirrored in the proteome, we applied label-free proteomics to the iPSC lines used in our previous study (Madsen et al., 2019). Approximately 4600 protein ratios were obtained for both heterozygous versus wild-type and homozygous versus wild-type iPSC comparisons, as estimated using a novel Bayesian approach based on the Markov Chain Monte Carlo (MCMC) method (Robin et al., 2019 preprint). In contrast to other algorithms, the MCMC method generates an error estimate alongside each protein concentration that permits a more confident determination of proteins with the most robust differential expression. The number of differentially expressed proteins correlated with *PIK3CA^H1047R^* allele dosage, with 54 and 258 differentially expressed proteins in *PIK3CA^WT/H1047R^* and *PIK3CA^H1047R/H1047R^* cells, respectively (Fig. 1F, Table S9,S10). Of these, 27 proteins were differentially expressed in both heterozygous and homozygous *PIK3CA^H1047R^* iPSCs (Table S11), with 16 changing in opposite directions (Fig. 1F). There was a strong correlation between differentially expressed proteins and corresponding transcripts in *PIK3CA^H1047R/H1047R^* iPSCs (Fig. S2A,B), but not in heterozygous mutants (Fig. S2C,D). As for the relatively weak correlation seen between transcriptomic experiments for heterozygous cells, this likely reflects the small magnitude of gene expression changes induced by heterozygous *PIK3CA^H1047R^* (Fig. 1B; Fig. S1C).

Collectively, these findings corroborate the existence of a threshold of PI3K pathway activity that determines the large majority of gene expression changes in *PIK3CA^H1047R/H1047R^* iPSCs in a near-binary manner. Although deeper sequencing did reveal statistically significant gene expression changes in heterozygous iPSCs, and although these changes may contribute to growth-related phenotypes in PROS when sustained across development, effect sizes were modest and more variable. Similar findings in heterozygous MEFs suggest that this may be generalisable to differentiated cell types, irrespective of species. This consolidates the view that only homozygosity for *PIK3CA^H1047R^* results in robust and widespread transcriptional changes in otherwise normal diploid cells, arguing against a universal ‘butterfly’ effect of heterozygosity based on studies of a genetically abnormal breast epithelial cell line (Hart et al., 2015; Kiselev et al., 2015).

### *PIK3CA^H1047R/H1047R^* iPSCs show evidence of signalling ‘rewiring’

We previously demonstrated a graded increase in AKT (S473) phosphorylation across heterozygous and homozygous *PIK3CA^H1047R^* iPSCs (Madsen et al., 2019). To assess in more detail whether the near-binary gene expression difference between heterozygous and homozygous *PIK3CA^H1047R^* cells is underpinned by corresponding differences in indices of PI3K pathway activation, we profiled phosphorylation of a wider repertoire of pathway components using reverse phase phosphoprotein array (RPPA) technology.

**Fig. 2.**
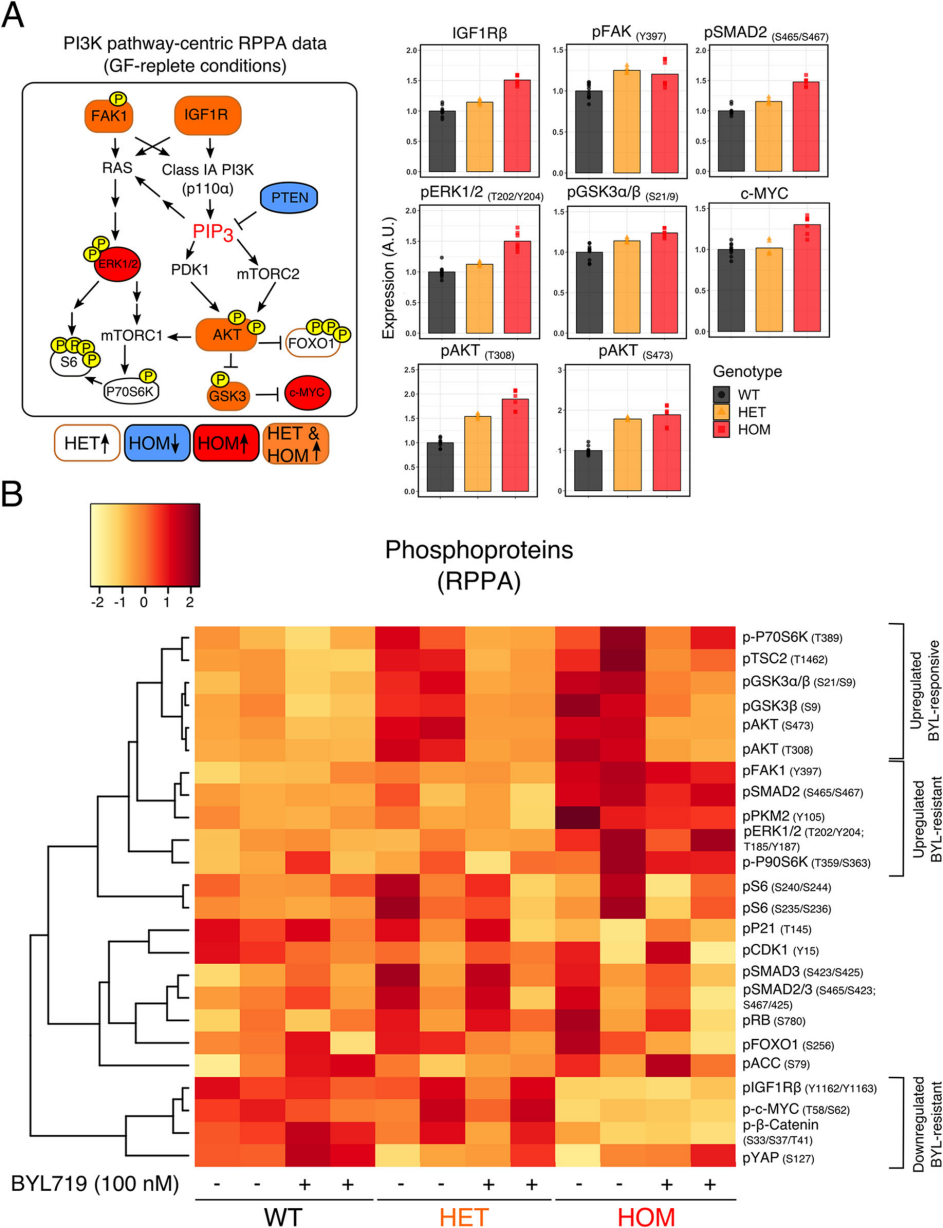
**RPPA of *PIK3CA^WT/H1047R^* (HET) and *PIK3CA^H1047R/H1047R^* (HOM) human iPSCs.** (A) Left: diagram of PI3K pathway-related phosphorylated proteins, with colour code used to signify differentially expressed targets in *PIK3CA^H1047R^* mutant iPSCs versus isogenic wild-type controls. Colour-coded targets were significant at an FDR of ≤0.05 (Benjamini-Hochberg). Right: barplots show representative examples of differentially expressed protein targets, revealing relatively modest quantitative changes. Phosphorylated proteins were normalised to the corresponding total protein when available. The data are based on ten wild-type cultures (three clones), five *PIK3CA^WT/H1047R^* cultures (three clones) and seven *PIK3CA^H1047R/H1047R^* cultures (two clones) as indicated. See also Fig. S3A. (B) Unsupervised hierarchical clustering based on target-wise correlations of RPPA data from wild-type (WT), *PIK3CA^WT/H1047R^* (HET) and *PIK3CA^H1047R/H1047R^* (HOM) iPSCs following short-term growth factor removal (1 h), ±100 nM BYL719 (PI3Kα inhibitor) for 24 h. The data are from two independent experiments, each performed using independent clones. For each row, the colours correspond to Fast Green-normalised expression values in units of s.d. (*z*-score) from the mean (centred at 0) across all samples (columns). Groups of phosphorylated proteins exhibiting a consistent expression pattern in BYL719-treated *PIK3CA^H1047R/H1047R^* iPSCs are specified. See also Fig. S3B.

Changes in protein phosphorylation were surprisingly modest, with the largest change a twofold increase in AKT phosphorylation (on S473 and T308) in *PIK3CA^H1047R/H1047R^* cells. Contrasting with the near-binary response seen at the transcriptional level, heterozygous and homozygous *PIK3CA^H1047R^* expression generally produced graded phosphorylation of PI3K pathway components, with slightly higher levels in homozygous iPSCs (Fig. 2A). None of the mutant genotypes showed consistently increased phosphorylation of the mTORC1 target P70S6K or its downstream substrate S6 (Fig. S3A), perhaps reflecting saturation at this level of the pathway due to other stimuli for mTORC1 in the complete culture medium (e.g. amino acids) (Valvezan and Manning, 2019). When deprived of growth factors for 1 h before RPPA profiling, both heterozygous and homozygous mutants exhibited increased P70S6K phosphorylation, whereas S6 phosphorylation remained similar to wild-type cells (Fig. 2B).

Inhibition of PI3Kα activity with the PI3Kα-selective inhibitor BYL719 for 24 h fully reversed canonical PI3K signalling-related changes in the phosphorylation of downstream proteins, including AKT, GSK3, FOXO1, TSC2 and P70S6K (Fig. 2B). Consistent with these signalling changes, we previously showed that the same dose of BYL719 (100 nM) abolishes the increased tolerance to growth factor deprivation-induced death conferred by heterozygous or homozygous *PIK3CA^H1047R^* in iPSCs (Madsen et al., 2019). Despite its effects on the primary PI3K signalling cascade, PI3Kα inhibition failed to reverse other changes observed in *PIK3CA^H1047R/H1047R^* iPSCs, including increased phosphorylation of SMAD2 and ERK1/2, and increased expression of c-MYC and IGF1R (Fig. 2B; Fig. S3B). This suggests signalling rewiring in *PIK3CA^H1047R/H1047R^* iPSCs that is partially resistant to relatively short-term inhibition of the inducing stimulus.

### Pathway and network analyses implicate NODAL/TGFβ signalling in *PIK3CA^H1047R^* allele dose-dependent stemness

**Fig. 3.**
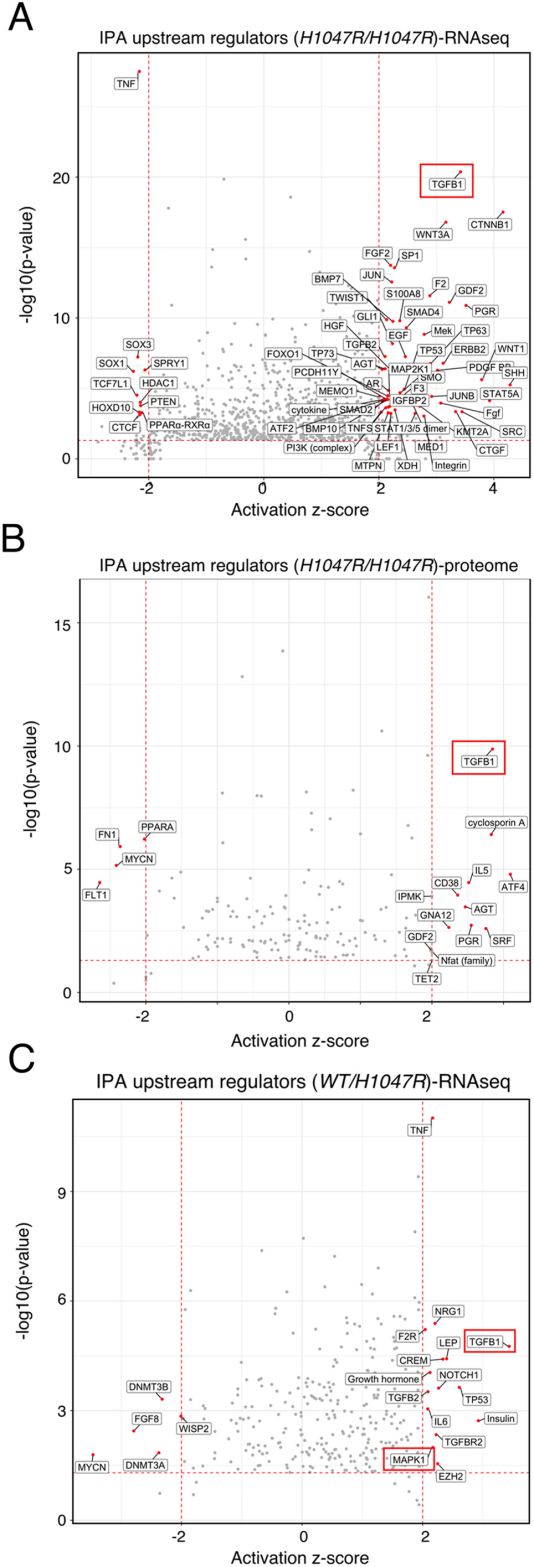
**IPAs predict activation of TGFβ signalling in heterozygous and homozygous *PIK3CA^H1047R^* iPSCs.** (A) IPA of upstream regulators using the list of the top 2000 upregulated and top 2000 downregulated mRNA transcripts in *PIK3CA^H1047R/H1047R^* iPSCs (for RNA-seq details, see Fig. 1A,B). Red points signify transcripts with an absolute predicted activation *z*-score of >2 and overlap *P*<0.001 (Fisher's Exact Test). The red rectangle highlights the most significant upstream regulator, TGFβ1. This analysis does not differentiate between NODAL, TGFβ1 and Activin, which all act through the same pathway in hPSCs. (B) As in A but using the list of differentially expressed proteins identified by total proteomics and red-colouring targets with a predicted activation *z*-score of >2 and overlap *P*<0.05 (Fisher's Exact Test). (C) As in A but using the list of differentially expressed transcripts in *PIK3CA^WT/H1047R^* iPSCs and red-colouring upstream regulators with an absolute predicted bias-corrected *z* score of >2 and overlap *P*<0.05 (Fisher's Exact Test). Red rectangles highlight the two upstream regulators (TGFβ1 and MAPK1) with an absolute predicted bias-corrected *z* score of >2 that remained significant (overlap *P*<0.05) when the analysis was repeated using the list of shared and concordant differentially expressed genes (*N*=180) in heterozygous and homozygous *PIK3CA^H1047R^* iPSCs versus wild-type controls.

Pathway and network analyses were next applied to proteomic and transcriptomic data to identify candidate mechanism(s) mediating *PIK3CA^H1047R^* allele dose-dependent stemness. Consistent with our previous study (Madsen et al., 2019), TGFβ1 was again the most significant predicted upstream activator according to Ingenuity Pathway Analysis (IPA) of the top 2000 upregulated and top 2000 downregulated transcripts in *PIK3CA^H1047R/H1047R^* iPSCs (Fig. 3A). TGFβ1 was also the most significant upstream activator predicted by analysis of *PIK3CA^H1047R/H1047R^* proteomic data (Fig. 3B). This is consistent with strong *NODAL* mRNA upregulation and increased pSMAD2 (S465/S467) in *PIK3CA^H1047R/H1047R^* iPSCs in this study (Table S6 and RPPA data in Fig. 2, respectively).

Although *PIK3CA^WT/H1047R^* iPSCs showed ∼tenfold fewer differentially expressed genes than homozygous iPSC cells, IPA in heterozygous iPSCs also revealed multiple TGFβ pathway-related stimuli among predicted upstream activators (Fig. 3C). Moreover, TGFβ1 was predicted to be one of only two significant upstream activators when analysis was performed on genes concordantly differentially expressed (*N*=180) in *PIK3CA^H1047R^* mutant iPSCs versus wild-type controls (Fig. 3C; Table S12).

The other significant upstream regulator common to heterozygous and homozygous *PIK3CA^H1047R^* iPSCs was MAPK1 (also known as ERK2), consistent with RPPA findings and immunoblot evidence of increased ERK kinase phosphorylation in *PIK3CA^H1047R^* mutant iPSCs (Madsen et al., 2019; Fig. 2A; Fig. S3A). The significance of predicted TGFβ activation in heterozygous *PIK3CA^H1047R^* iPSCs (overlap *P*-value=1.7e-05) was much lower than in homozygous (overlap *P*=4.3e-21) mutants. This is in keeping with the much lower effect size in heterozygous cells, and consistent with a critical role for the NODAL/TGFβ pathway in mediating the allele dose-dependent effect of *PIK3CA^H1047R^* in human iPSCs.

**Fig. 4.**
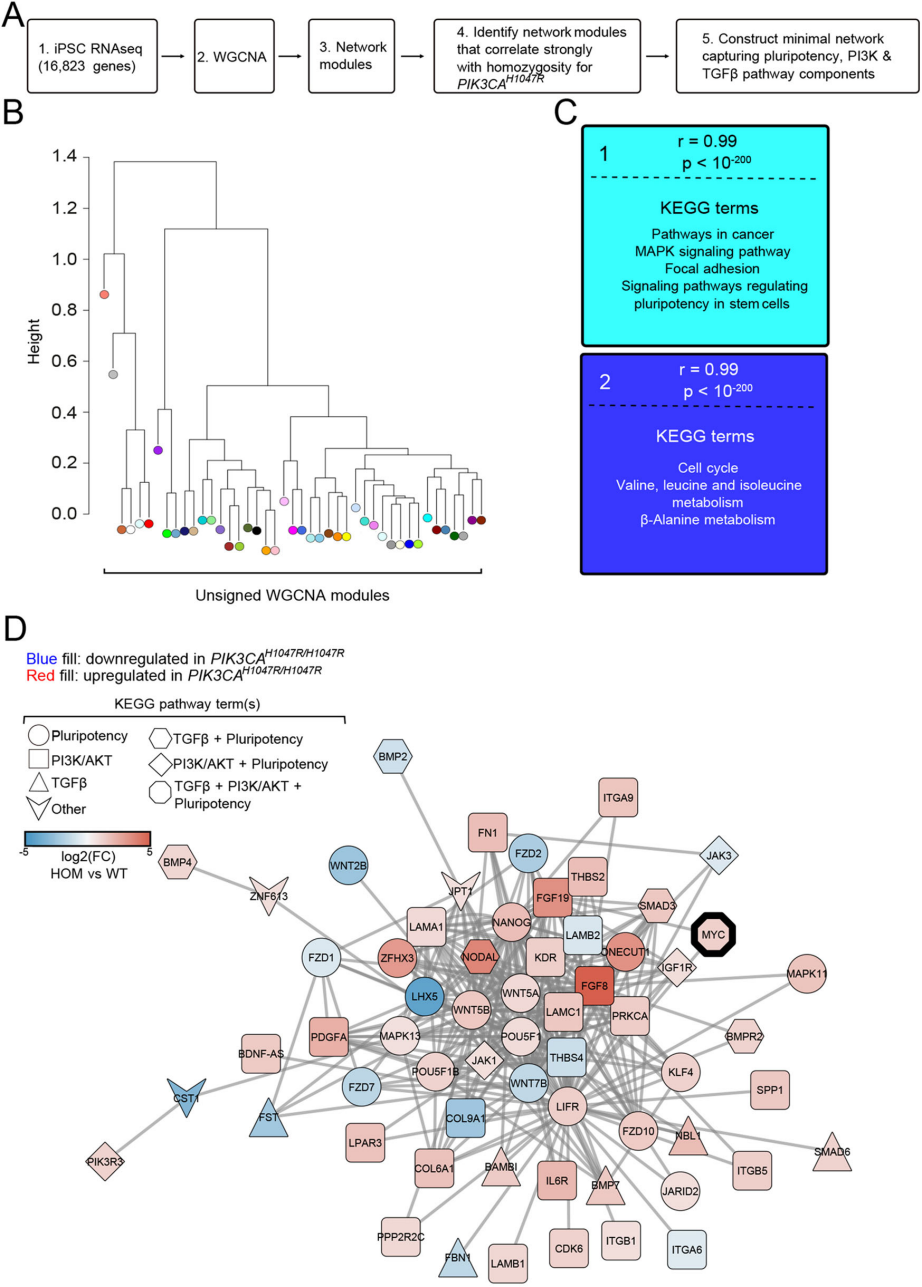
**WGCNA identifies links among pluripotency components, TGFβ and PI3K signalling.** (A) Schematic of the WGCNA workflow and subsequent data selection for visualisation. (B) Unsigned WGCNA modules identified using the list of transcripts expressed in wild-type, *PIK3CA^WT/H1047R^* and *PIK3CA^H1047R/H1047R^* iPSCs (for RNA-seq details, see Fig. 1B). (C) The two network modules with genes with a module membership that correlated strongest with differential expression in homozygous *PIK3CA^H1047R^* iPSCs. The colour of each module corresponds to its colour in the module dendrogram in B. Representative KEGG pathways with significant enrichment in each gene network module are listed (hypergeometric test with two-sided uncorrected *P*<0.05). (D) The minimal network connecting KEGG pluripotency, PI3K/AKT and TGFβ pathway components within the turquoise gene network module. Fill colour and shape are used to specify direction of differential mRNA expression in *PIK3CA^H1047R/H1047R^* iPSCs and pathway membership, respectively. Fill colour saturation represents gene expression fold change (FC; log2) in *PIK3CA^H1047R/H1047R^* (HOM) versus wild-type (WT) iPSCs. MYC is highlighted as the only network component intersecting all three KEGG pathways, suggesting it may comprise a key mechanistic link in the observed phenotype.

To complement IPA analysis, which is based on highly curated proprietary datasets, we undertook non-hypothesis-based weighted gene correlation network analysis (WGCNA) – a network-based data reduction method that seeks to determine gene correlation patterns across multiple samples, irrespective of the function of individual genes (Langfelder and Horvath, 2008). Using all transcripts expressed in wild-type, heterozygous and homozygous *PIK3CA^H1047R^* iPSCs (Fig. 4A), this analysis returned 43 modules (or clusters) of highly interconnected genes (Fig. 4B). Of the two modules with the highest correlation with the homozygous trait, one showed enrichment for several Kyoto Encyclopedia of Genes and Genomes (KEGG) pathway terms relevant to stemness of *PIK3CA^H1047R/H1047R^* iPSCs, notably including ‘signalling pathways regulating pluripotency in stem cells’ (Fig. 4C).

Given previous evidence of strong activation of NODAL/TGFβ signalling in homozygous mutant cells, we next constructed the minimal network of differentially expressed genes in *PIK3CA^H1047R/H1047R^* iPSCs that linked pluripotency, PI3K and TGFβ signalling pathways (Fig. 4D). This approach allowed us to navigate the signalling rewiring and to link strong PI3K pathway activation, stemness and NODAL/TGFβ signalling in an unbiased manner. Indeed, the resulting network exhibited high interconnectivity, with multiple shared nodes across all three pathways, suggesting close crosstalk between PI3K and NODAL/TGFβ signalling in stemness regulation. That most nodes represented genes with increased expression in homozygous mutants strengthens the notion that strong oncogenic PI3Kα activation stabilises the pluripotency network in human iPSCs. The MYC oncogene stood out as the only network node intersecting with all three signalling pathways, suggesting it may comprise a key mechanistic link in the observed phenotype.

### Inhibition of NODAL/TGFβ signalling destabilises the pluripotency gene network in *PIK3CA^H1047R/H1047R^* iPSCs

NODAL/TGFβ signalling plays a critical role in pluripotency regulation (Vallier et al., 2005; Mesnard et al., 2006; Xu et al., 2008), and a differentiation-resistant phenotype has been reported in *NODAL*-overexpressing iPSCs (Vallier et al., 2004). Together with increased *NODAL* expression in homozygous *PIK3CA^H1047R^* iPSCs and computational identification of enhanced NODAL/TGFβ pathway activity in PI3K-driven ‘constitutive’ stemness (Madsen et al., 2019 and current study), this led us to hypothesise that strong PI3Kα-dependent induction of *NODAL* underlies establishment of the differentiation-resistant phenotype of these cells. Specifically, we hypothesised that autocrine NODAL enhances NODAL/TGFβ signalling in *PIK3CA^H1047R/H1047R^* iPSCs, with resulting increased *NANOG* expression ‘locking’ the cells in perpetual stemness (Xu et al., 2008).

Testing this hypothesis in iPSCs is challenging for biological and technical reasons, including lack of specific pharmacological inhibitors of NODAL, and difficulty in detecting subtle early phenotypic consequences of partial destabilisation of the iPSC pluripotency gene regulatory network. Moreover, the widely adopted maintenance medium and coating substrate we used for cell culture both contain TGFβ ligands (Vukicevic et al., 1992; Chen et al., 2011), which may mask effects of *NODAL* repression by PI3Kα-specific inhibition. We previously found that treatment of *PIK3CA^H1047R/H1047R^* iPSCs in this ‘complete’ maintenance medium with 500 nM BYL719 reduces *NODAL* mRNA expression within 24 h, but has no discernible effect on increased *NANOG* mRNA levels (Madsen et al., 2019).

**Fig. 5.**
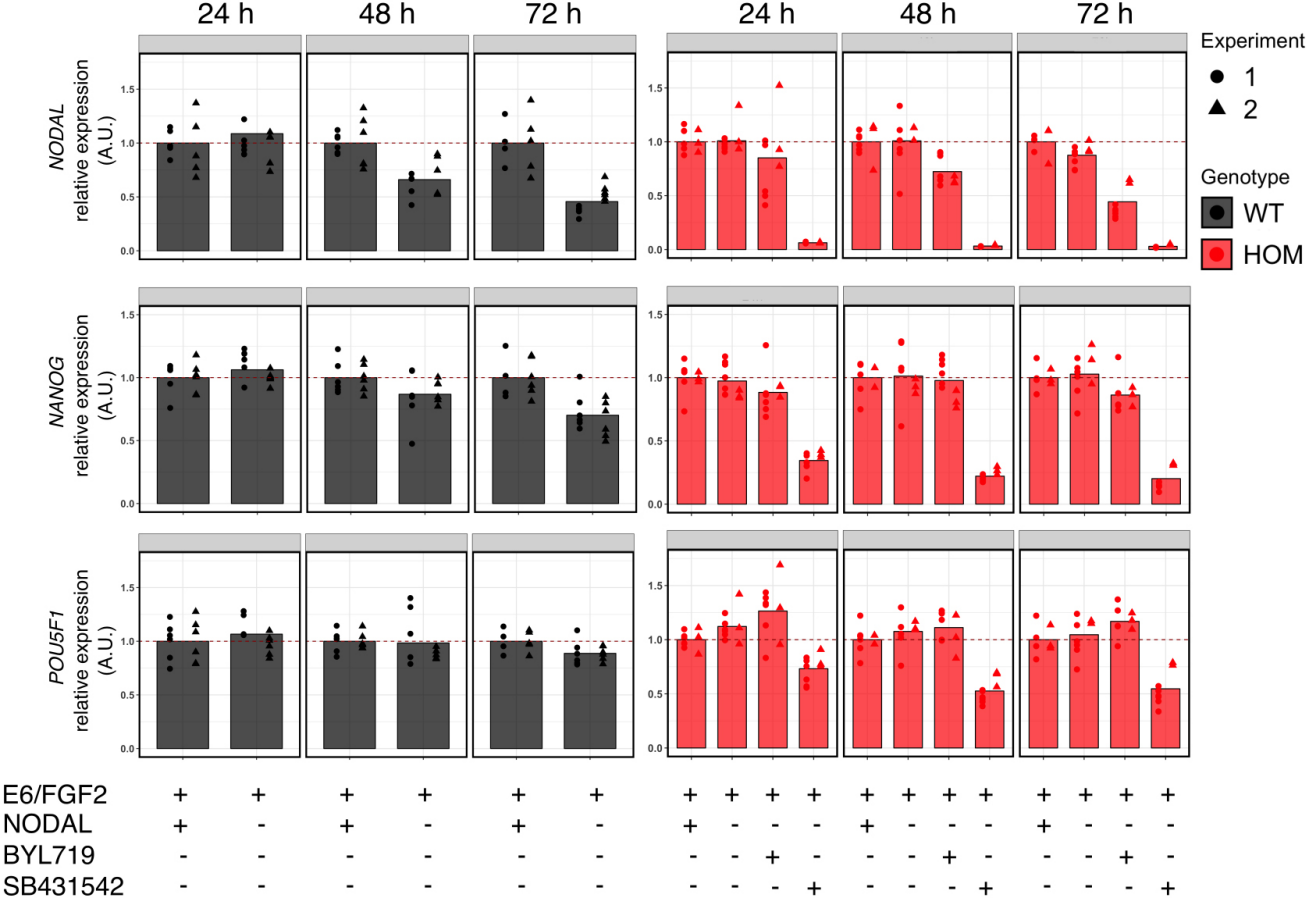
**NODAL/TGFβ signalling-dependent regulation of stemness in *PIK3CA^H1047R/H1047R^* iPSCs.** Gene expression time course of *NODAL*, *NANOG* and *POU5F1* in wild-type (WT) or *PIK3CA^H1047R/H1047R^* iPSCs following the indicated treatments for 24 h, 48 h or 72 h. E6/FGF2, essential 6 medium supplemented with 10 ng ml^−1^ basic fibroblast growth factor 2 (FGF2). BYL719 is a PI3Kα-selective inhibitor used at 250 nM. SB431542 is a specific inhibitor of the NODAL type I receptors ALK4/7 and the TGFβ type I receptor ALK5; used at 5 µM. When indicated, cultures were supplemented with 100 ng ml^−1^ NODAL. The data are from two independent experiments, with each treatment applied to triplicate cultures of three wild-type and two homozygous iPSC clones. To aid interpretation, gene expression values are normalised to the E6/FGF2 condition within each genotype and time point. An alternative visualisation that illustrates the differential expression of *NODAL* and *NANOG* between mutant and wild-type cells is shown in Fig. S4A. For analysis of additional lineage markers, see Fig. S4B. For representative micrographs of *PIK3CA^H1047R/H1047R^* iPSCs exposed to the different treatments, see Fig. S5. A.U., arbitrary units.

To minimise confounding effects of exogenous TGFβ ligands, we prepared medium with and without recombinant NODAL supplementation, and assessed expression of *NODAL* and *NANOG* as surrogate markers of stemness over 72 h of culture. We also reduced the BYL719 concentration to 250 nM given the increased iPSC toxicity observed with 500 nM BYL719 (Madsen et al., 2019); and pilot experiments (not shown) in which 24 h treatment with 250 nM but not 100 nM BYL719 in complete medium reduced *NODAL* mRNA expression in *PIK3CA^H1047R/H1047R^* iPSC clones. Within 48 h, exclusion of NODAL from the medium resulted in the expected downregulation of *NODAL* and *NANOG* expression in wild-type iPSCs, and this was greater still at 72 h (Fig. 5; Fig. S4A). However, in *PIK3CA^H1047R/H1047R^* iPSCs, *NODAL* removal had no effect on the increased *NODAL* and *NANOG* expression (Fig. 5; Fig. S4A), in line with a self-sustained stemness phenotype. Exposure of NODAL-free *PIK3CA^H1047R/H1047R^* cultures to 250 nM BYL719 had a visible colony growth-inhibitory effect (Fig. S5) and decreased *NODAL* expression within 24 h, and this continued to decrease subsequently (Fig. 5). This is consistent with the known ability of NODAL to control its own expression through a feed-forward loop (Hill, 2018). However, despite a 55% reduction in *NODAL* mRNA after 72 h, little effect on *NANOG* expression was seen (Fig. 5). This may reflect the short time course studied (to avoid confounding effect of passaging), or the exquisite sensitivity of iPSCs to residual upregulation of *NODAL* in homozygous *PIK3CA^H1047R^* iPSCs. This may be compounded by residual low levels of TGFβ-like ligands in the coating substrate, or possibly by increased expression of two other TGFβ superfamily ligands, *GDF3* and *TGFB2*, observed in homozygous mutant cells (Table S6).

To confirm that NODAL/TGFβ signalling is required for the maintenance of stemness in *PIK3CA^H1047R/H1047R^* iPSCs, the cells were treated with SB431542 – a specific inhibitor of TGFβ and NODAL type I receptors (Inman et al., 2002). This completely repressed *NODAL* expression within 24 h, accompanied by downregulation of *NANOG* and *POU5F1* expression (Fig. 5). Given that the pluripotent stem cell state is stabilised by an autoregulatory feedforward interaction between these transcription factors, their downregulation is expected to result in altered expression of multiple stemness and differentiation markers. Confirming this, we used a lineage-specific gene expression array to demonstrate a similar reduction in mRNA expression of several other well-established stemness markers (*MYC*, *FGF4* and *GDF3*), which were upregulated in *PIK3CA^H1047R/H1047R^* iPSCs at baseline (Table S6), performing the analysis after 48 h of NODAL/TGFβ pathway inhibition (Fig. S4B). Despite the short treatment, we also found evidence for the expected neuroectoderm induction upon inhibition of the NODAL/TGFβ pathway (Vallier et al., 2004, 2009), reflected by increased expression of *CDH9*, *MAP2* and *PAPLN* (Fig. S4B).

Collectively, these data suggest that the stemness phenotype of *PIK3CA^H1047R/H1047R^* iPSCs is mediated by self-sustained NODAL/TGFβ signalling, most likely through PI3K dose-dependent increase in *NODAL* expression, and that this is amenable to reversal through inhibition of the NODAL/TGFβ pathway but not of PI3Kα itself.

## DISCUSSION

*PIK3CA^H1047R^* is the most common activating *PIK3CA* mutation in human cancers and in PROS (Madsen et al., 2018). We recently found that *PIK3CA*-associated cancers often harbour multiple mutated *PIK3CA* copies, and demonstrated that homozygosity but not heterozygosity for *PIK3CA^H1047R^* leads to self-sustained stemness in hPSCs (Madsen et al., 2019). High-depth transcriptomics in this study confirmed that heterozygosity for *PIK3CA^H1047R^* induces significant but very modest transcriptional changes; observed both in CRISPR-edited hPSCs with long-term *PIK3CA^H1047R^* expression and in mouse embryonic fibroblasts (MEFs) upon acute *PIK3CA^H1047R^* induction by *Cre*, with canonical PI3K pathway activation seen in both cases (this study and Berenjeno et al., 2017; Moniz et al., 2017; Madsen et al., 2019). Similarly, hPSCs with heterozygous expression of *PIK3CA^E418K^*, a ‘non-hotspot’ mutation, were transcriptionally indistinguishable from their isogenic wild-type controls. However, in contrast to the mild transcriptional consequences of these heterozygous variants, homozygosity for *PIK3CA^H1047R^* was associated with differential expression of nearly a third of the hPSC transcriptome, suggesting widespread epigenetic reprogramming. This near-binary response is not a consequence of a similar quantitative difference in PI3K pathway activation, as assessed by phosphoprotein profiling, which instead showed a relatively modest and graded increase in homozygous versus heterozygous *PIK3CA^H1047R^* hPSCs. This implies that the apparent PI3K signalling threshold that determines the cellular response in hPSCs is ‘decoded’ distally to the canonical pathway activation.

Using a combination of computational analyses and targeted experiments, this study further provides evidence for self-sustained NODAL/TGFβ pathway activation as the main mechanism through which *PIK3CA^H1047R^* homozygosity ‘locks’ hPSCs in a differentiation-resistant state that has become independent of the driver mutation and the associated PI3K pathway activation. We suggest that homozygosity but not heterozygosity for *PIK3CA^H1047R^* promotes sufficient NODAL/TGFβ pathway activity to induce increased *NODAL* and downstream *NANOG* expression to levels that stabilise the stem cell state, yet are not high enough to tip the balance towards mesendoderm differentiation (Madsen et al., 2019). Exactly how PI3K activation regulates *NODAL* expression remains unknown. A potential mechanism involves increased expression of the stem cell reprogramming factor MYC, which was observed at both mRNA and protein levels in homozygous but not heterozygous *PIK3CA^H1047R^* iPSCs. Furthermore, MYC was the only node in the WGCNA-based network of pluripotency, PI3K and TGFβ pathway components that was shared by all three pathways (Fig. 4D). MYC has previously been shown to exert oncogenic effects that depend on a sharp threshold of MYC expression, reminiscent of the effects we observe for allele dose-dependent *PIK3CA* activation (Murphy et al., 2008). Elevated MYC has also been shown to allow *PIK3CA^H1047R^*-induced murine breast cancers to become independent of continuous *PIK3CA^H1047R^* expression (Liu et al., 2011).

Stabilisation of the stemness phenotype in hPSCs by strong genetic PI3K pathway activation may be generalisable beyond this model system. BYL719 (alpelisib; Novartis), the PI3Kα-selective inhibitor used in our cellular studies, was recently approved for use in combination with anti-oestrogen therapy in oestrogen receptor-positive breast cancers (André et al., 2019). In a separate study focusing on human breast cancer, we have described the use of computational analyses to demonstrate a strong positive relationship between a transcriptomically derived PI3K activity score, stemness gene expression and tumour grade in breast cancer (Madsen et al., 2020 preprint). Previous reports have suggested a role for NODAL in driving breast cancer stemness and aggressive disease (Bar-Eli, 2012; Margaryan et al., 2019), with potential links to mTORC1 activation (Katsuno et al., 2019; Jewer et al., 2020). Our findings that BYL719 fails to fully reverse the increased *NODAL* and stemness gene expression in homozygous *PIK3CA^H1047R^* iPSCs suggests that inhibition of TGFβ signalling as a pro-differentiation therapy warrants investigation as a co-therapy with PI3K inhibitors in breast tumours with strong PI3K pathway activation. The lack of widespread transcriptional changes upon heterozygous expression of mutant *PIK3CA* in otherwise genetically normal cell models may explain the low oncogenicity of this genotype in isolation *in vivo*.

Finally, our observations are important for future studies seeking to model human PIK3CA-related diseases. The modest changes observed in heterozygous *PIK3CA^H1047R^* cells, in sharp contrast to the radical transcriptional alterations in homozygous cells, emphasise the importance of careful allele dose titration when artificial overexpression systems are used to model disorders caused by genetic *PIK3CA* activation. Our findings in heterozygous cells are also a reminder that very small effect sizes in cellular systems may summate and result in major human phenotypes over a life course. That such minor changes are found in a cellular study of a rare and severe disorder emphasises the challenges of modelling much more subtle disease susceptibility conferred by GWAS-detected genetic associations, in which cellular effect sizes are likely to be smaller still.

## MATERIALS AND METHODS

All cell lines used in this study are listed in Table S1. Unless stated otherwise, standard chemicals were acquired from Sigma-Aldrich, with details for the remaining reagents included in Tables S2, S3 and S4.

### iPSC culture and treatments

#### Maintenance

The derivation of the iPSC lines, including associated ethics statements, has been described previously (Madsen et al., 2019). All lines were grown at 37°C and 5% CO_2_ in Essential 8 Flex (E8/F) medium on Geltrex-coated plates, in the absence of antibiotics. For maintenance, cells at 70-90% confluency were passaged as aggregates with ReLeSR, using E8 supplemented with RevitaCell (E8/F+R) during the first 24 h to promote survival. A detailed version of this protocol is available via protocols.io (doi: dx.doi.org/10.17504/protocols.io.4rtgv6n).

All cell lines were tested negative for mycoplasma and genotyped routinely to rule out cross-contamination during prolonged culture. Short tandem repeat profiling was not performed. All experiments were performed on cells within ten passages after thawing.

#### Collection for RNA sequencing and total proteomics

For RNA sequencing and total proteomics, subconfluent cells were fed fresh E8/F 3 h before snap-freezing on dry ice and subsequent RNA or protein extraction. Relative to the results in Madsen et al. (2019), the current transcriptomic data of PIK3CAH1047R were obtained more than 6 months following the first study, with cells at different passages, and were thus independent from one another. Moreover, sample collection for the second transcriptomics experiment was conducted over 3 days according to a block design, thus allowing us to determine transcriptional differences that are robust to biological variability.

#### Cell lysate collection for RPPA

For RPPA in growth factor-replete conditions, cells were fed fresh E8/F 3 h before collection. To assess variability due to differences in collection timing, clones from each iPSC genotype were collected on each one of 3 days according to a block design, giving rise to a total of 22 cultures. To test the effect of the PI3Kα-specific inhibitor BYL719, cells were treated with 100 nM drug (or DMSO only as control treatment) for 24 h and exposed to growth factor removal within the last hour before collection. All cells were washed in Dulbecco's PBS (without calcium and magnesium) before collection to rinse off residual proteins and cell debris.

#### NODAL/TGFβ signalling studies

Wild-type or homozygous PIK3CAH1047R iPSCs were seeded in 12-well plates all coated with Geltrex from the same lot (2052962; diluted in Dulbecco's modified Eagle's medium/F12, lot RNBH0692). Cells were processed for seeding at a ratio of 1:15 according to the standard maintenance protocol. One day after seeding, individual treatments were applied to triplicate wells. Briefly, cells were first washed twice with 2 ml and 1 ml of Dulbecco's PBS to remove residual growth factors. The base medium for individual treatments was Essential 6 supplemented with 10 ng ml^−1^ heat-stable FGF2. This was combined with one of the following reagents or their diluent equivalents: 100 ng ml^−1^ NODAL (diluent, 4 mM HCl); 250 nM BYL719 (diluent, DMSO); and 5 µM SB431542 (diluent, DMSO). Cells were snap-frozen on dry ice after 24, 48 and 72 h following a single Dulbecco's PBS wash. Individual treatments were replenished daily at the same time of day to limit temporal confounders.

### Mouse embryonic fibroblast culture

The derivation and culture of the wild-type and PIK3CAWT/H1047R MEFs used in this study have been reported previously (Moniz et al., 2017). Cell pellets were collected on dry ice 48 h after induction of heterozygous PIK3CAH1047R expression, without prior starvation.

### RNA-seq

Induced pluripotent stem cell lysates were collected in QIAzol and processed for RNA extraction with the DirectZol Kit as per the manufacturer's instructions. The final RNA was subjected to quantification and quality assessment on an Agilent Bioanalyzer using the RNA 6000 Nano Kit, confirming that all samples had a RNA integrity number (RIN) score of 10. For PIK3CAH1047R iPSCs and corresponding wild types, an Illumina TruSeq Stranded mRNA Library Prep Kit was used to synthesise 150-bp-long paired-end mRNA libraries, followed by sequencing on an Illumina HiSeq 4000, with an average depth of 70 million reads per sample. *PIK3CA^WT/E418K^* and isogenic control iPSCs were subjected to 50-bp-long single-end RNA-seq at an average depth of 20 million reads per sample.

MEF RNA was extracted using Qiagen's RNAeasy miniprep (with QIAshredder). All samples had a confirmed Agilent Bioanalyzer RIN score of 10. An Illumina TruSeq Unstranded mRNA kit was used to prepare 100-bp-long paired-end libraries, followed by Illumina HiSeq 2000 sequencing. Details of the subsequent data analyses (raw read mapping, counting, statistical testing, pathway and network analyses) are provided in supplementary Materials and Methods.

### Label-free total proteomics

#### Sample preparation

Cells were cultured to subconfluence in Geltrex-coated T175 flasks, and protein was harvested by lysis in 3 ml modified RIPA buffer [50 mM Tris-HCl (pH 7.5), 150 mM NaCl, 1% NP-40, 0.5% Na-deoxycholate and 1 mM EDTA] supplemented with phosphatase inhibitors (5 mM β-glycerophosphate, 5 mM NaF, 1 mM Na3VO4) and protease inhibitors (Roche cOmplete ULTRA Tablets, EDTA-free). The lysates were sonicated on ice (4×10 s bursts, amplitude, 60%; Bandelin Sonopuls HD2070 sonicator) and spun down for 20 min at 4300 ***g***. Ice-cold acetone was added to the supernatant to achieve a final concentration of 80% acetone, and protein was left to precipitate overnight at −20°C. Precipitated protein was pelleted by centrifugation at 2000 ***g*** for 5 min and solubilised in 6 M urea, 2 M thiourea and 10 mM HEPES (pH 8.0). Protein was quantified using the Bradford assay and 8 mg of each sample was reduced with 1 mM dithiothreitol, alkylated with 5 mM chloroacetamide and digested with endopeptidase Lys-C (1:200 v/v) for 3 h. Samples were diluted to 1 mg ml^−1^ protein using 50 mM ammonium bicarbonate and incubated overnight with trypsin (1:200 v/v). Digested samples were acidified and urea removed using SepPak C18 cartridges. Peptides were eluted, and an aliquot of 100 μg set aside for total proteome analysis. The peptides were quantified using the Pierce quantitative colorimetric peptide assay. The equalised peptide amounts were lyophilised and resolubilised in 2% acetonitrile and 1% trifluoroacetic acid in order to achieve a final 2 μg on-column peptide load.

#### Mass spectrometry data acquisition

All spectra were acquired on an Orbitrap Fusion Tribrid mass spectrometer (Thermo Fisher Scientific) operated in data-dependent mode coupled to an EASY-nLC 1200 liquid chromatography pump (Thermo Fisher Scientific) and separated on a 50 cm reversed phase column (Thermo Fisher Scientific, PepMap RSLC C18, 2 µM, 100A, 75 µm×50 cm). Proteome samples (non-enriched) were eluted over a linear gradient ranging from 0-11% acetonitrile over 70 min, 11-20% acetonitrile for 80 min, 21-30% acetonitrile for 50 min, 31-48% acetonitrile for 30 min, followed by 76% acetonitrile for the final 10 min with a flow rate of 250 nl/min.

Survey-full scan mass spectrometry spectra were acquired in the Orbitrap at a resolution of 120,000 from m/z 350-2000, automated gain control (AGC) target of 4×10^5^ ions, and maximum injection time of 20 ms. Precursors were filtered based on charge state (≥2) and monoisotopic peak assignment, and dynamic exclusion was applied for 45 s. A decision tree method allowed fragmentation for ion trap MS2 via electron transfer dissociation (ETD) or higher-energy collision dissociation (HCD), depending on charge state and m/z. Precursor ions were isolated with the quadrupole set to an isolation width of 1.6 m/z. MS2 spectra fragmented by ETD and HCD (35% collision energy) were acquired in the ion trap with an AGC target of 1e4. Maximum injection time for HCD and ETD was 80 ms for proteome samples. Details of the subsequent data analyses (FASTA file generation and mass spectrometry searches) are provided in supplementary Materials and Methods.

### Reverse phase protein array

For RPPA, snap-frozen cells were lysed in ice-cold protein lysis buffer containing 50 mM HEPES, 150 mM NaCl, 1.5 mM MgCl2, 10% (v/v) glycerol, 1% (v/v) Triton X-100, 1 mM EGTA, 100 mM NaF, 10 mM Na4P2O7, 2 mM Na3VO4 (added fresh), a 1× EDTA-free protease inhibitor tablet and a 1× PhosStop tablet. Protein concentrations were measured using the Bio-Rad DC protein assay, and all concentrations were adjusted to 1 mg ml^−1^ with lysis buffer and 1× SDS sample buffer [10% glycerol, 2% SDS and 62.5 mM Tris-HCl (pH 6.8)] supplemented with 2.5% β-mercaptoethanol.

The protein lysates were processed for slide spotting and antibody incubations as described previously (Macleod et al., 2017). Briefly, a four-point dilution series was prepared for each sample and printed in triplicate on single pad Avid nitrocellulose slides (Grace Biolabs) consisting of eight arrays with 36×12 spots each. Next, slides were blocked and incubated in primary and secondary antibodies. The processed arrays were imaged using an Innopsys 710 slide scanner. Non-specific signals were determined for each slide by omitting the primary antibody incubation step. For normalisation, sample loading on each array was determined by staining with Fast Green dye and recording the corresponding signal at 800 nm. Details for all primary and secondary RPPA antibodies are included in Table S3. Details of all subsequent data analyses, including statistical testing and evaluation of antibody/assay performance, are provided in supplementary Materials and Methods.

### RT-qPCR

Cellular RNA was extracted as described above for RNA-seq, and 200 ng was used for cDNA synthesis with High-Capacity cDNA Reverse Transcription Kit (Thermo Fisher Scientific). Subsequent SYBR Green-based qPCRs were performed on 2.5 ng total cDNA. TaqMan hPSC Scorecards (384-well) were used according to the manufacturer's instructions with minor modifications. Further details on protocol modifications and all data analysis steps are provided in supplementary Materials and Methods. Details for all primers are included in Table S4.

### Statistical analyses

Bespoke statistical analyses are specified in the relevant sections above and in supplementary Materials and Methods. No statistical methods were used to predetermine sample size.

## Supplementary Material

Supplementary information

## References

[DMM048298C1] André, F., Ciruelos, E., Rubovszky, G., Campone, M., Loibl, S., Rugo, H. S., Iwata, H., Conte, P., Mayer, I. A., Kaufman, B. et al. (2019). Alpelisib for PIK3CA-mutated, hormone receptor-positive advanced breast cancer. *N. Engl. J. Med.* 380, 1929-1940. 10.1056/NEJMoa1813904 31091374

[DMM048298C2] Bar-Eli, M. (2012). Back to the embryonic stage: nodal as a biomarker for breast cancer progression. *Breast Cancer Res.* 14, 105. 10.1186/bcr3177 22643182 PMC3446330

[DMM048298C3] Berenjeno, I. M., Piñeiro, R., Castillo, S. D., Pearce, W., Mcgranahan, N., Dewhurst, S. M., Meniel, V., Birkbak, N. J., Lau, E., Sansregret, L. et al. (2017). Oncogenic PIK3CA induces centrosome amplification and tolerance to genome doubling. *Nat. Commun.* 8, 1773. 10.1038/s41467-017-02002-4 29170395 PMC5701070

[DMM048298C4] Bilanges, B., Posor, Y. and Vanhaesebroeck, B. (2019). PI3K isoforms in cell signalling and vesicle trafficking. *Nat. Rev. Mol. Cell Biol.* 20, 515-534. 10.1038/s41580-019-0129-z 31110302

[DMM048298C5] Boyer, L., Lee, T. I., Cole, M. F., Johnstone, S. E., Levine, S. S., Zucker, J. P., Guenther, M. G., Kumar, R. M., Murray, H. L., Jenner, R. G. et al. (2005). Core transcriptional regulatory circuitry in human embryonic stem cells. *Cell* 122, 947-956. 10.1016/j.cell.2005.08.020 16153702 PMC3006442

[DMM048298C6] Campbell, P. J., Getz, G., Korbel, J. O., Stuart, J. M., Jennings, J. L., Stein, L. D., Perry, M. D., Nahal-Bose, H. K., Ouellette, B., Li, F. and Constance, H. (2020). Pan-cancer analysis of whole genomes. *Nature* 578, 82-93. 10.1038/s41586-020-1969-6 32025007 PMC7025898

[DMM048298C7] Chambers, I., Colby, D., Robertson, M., Nichols, J., Lee, S., Tweedie, S. and Smith, A. (2003). Functional expression cloning of Nanog, a pluripotency sustaining factor in embryonic stem cells. *Cell* 113, 643-655. 10.1016/S0092-8674(03)00392-1 12787505

[DMM048298C8] Chambers, I., Silva, J., Colby, D., Nichols, J., Nijmeijer, B., Robertson, M., Vrana, J., Jones, K., Grotewold, L. and Smith, A. (2007). Nanog safeguards pluripotency and mediates germline development. *Nature* 450, 1230-1234. 10.1038/nature06403 18097409

[DMM048298C9] Chang, M. T., Asthana, S., Gao, S. P., Lee, B. H., Chapman, J. S., Kandoth, C., Gao, J. J., Socci, N. D., Solit, D. B., Olshen, A. B. et al. (2015). Identifying recurrent mutations in cancer reveals widespread lineage diversity and mutational specificity. *Nat. Biotechnol.* 34, 155-163. 10.1038/nbt.3391 26619011 PMC4744099

[DMM048298C10] Chen, G., Gulbranson, D. R., Hou, Z., Bolin, J. M., Ruotti, V., Probasco, M. D., Smuga-Otto, K., Howden, S. E., Diol, N. R., Propson, N. E. et al. (2011). Chemically defined conditions for human iPSC derivation and culture. *Nat. Methods* 8, 424-429. 10.1038/nmeth.1593 21478862 PMC3084903

[DMM048298C11] Darr, H. (2006). Overexpression of NANOG in human ES cells enables feeder-free growth while inducing primitive ectoderm features. *Development* 133, 1193-1201. 10.1242/dev.02286 16501172

[DMM048298C12] Fruman, D. A., Chiu, H., Hopkins, B. D., Bagrodia, S., Cantley, L. C. and Abraham, R. T. (2017). The PI3K pathway in human disease. *Cell* 170, 605-635. 10.1016/j.cell.2017.07.029 28802037 PMC5726441

[DMM048298C13] Hart, J. R., Zhang, Y., Liao, L., Ueno, L., Du, L., Jonkers, M., Yates, J. R. and Vogt, P. K. (2015). The butterfly effect in cancer: a single base mutation can remodel the cell. *Proc. Natl Acad. Sci. USA* 112, 1131-1136. 10.1073/pnas.1424012112 25583473 PMC4313835

[DMM048298C14] Hill, C. S. (2018). Spatial and temporal control of NODAL signaling. *Curr. Opin. Cell Biol.* 51, 50-57. 10.1016/j.ceb.2017.10.005 29153705

[DMM048298C15] Inman, G. J., Nicolás, F. J., Callahan, J. F., Harling, J. D., Gaster, L. M., Reith, A. D., Laping, N. J. and Hill, C. S. (2002). SB-431542 is a potent and specific inhibitor of transforming growth factor-beta superfamily type I activin receptor-like kinase (ALK) receptors ALK4, ALK5, and ALK7. *Mol. Pharmacol.* 62, 65-74. 10.1124/mol.62.1.65 12065756

[DMM048298C16] Ivanova, N. dobrin, R., Lu, R., Kotenko, I., Levorse, J., Decoste, C., Schafer, X., Lun, Y. and Lemischka, I. R. (2006). Dissecting self-renewal in stem cells with RNA interference. *Nature* 442, 533-538. 10.1038/nature04915 16767105

[DMM048298C17] Jewer, M. Lee, L., Leibovitch, M., Zhang, G., Liu, J., Findlay, S. D., Vincent, K. M., Tandoc, K., Dieters-Castator, D., Quail, D. F. et al. (2020). Translational control of breast cancer plasticity. *Nat. Commun.* 11, 2498. 10.1038/s41467-020-16352-z 32427827 PMC7237473

[DMM048298C18] Katsuno, Y., Meyer, D. S., Zhang, Z., Shokat, K. M., Akhurst, R. J., Miyazono, K. and Derynck, R. (2019). Chronic TGF-b exposure drives stabilized EMT, tumor stemness, and cancer drug resistance with vulnerability to bitopic mTOR inhibition. *Sci. Signal.* 12, eaau8544. 10.1126/scisignal.aau8544 30808819 PMC6746178

[DMM048298C19] Kiselev, V. Y., Juvin, V., Malek, M., Luscombe, N., Hawkins, P., Novère, N. L. and Stephens, L. (2015). Perturbations of PIP3 signalling trigger a global remodelling of mRNA landscape and reveal a transcriptional feedback loop. *Nucleic Acids Res.* 43, 9663-9679. 10.1093/nar/gkv1015 26464442 PMC4787766

[DMM048298C20] Langfelder, P. and Horvath, S. (2008). WGCNA: an R package for weighted correlation network analysis. *BMC Bioinformatics* 9, 559. 10.1186/1471-2105-9-559 19114008 PMC2631488

[DMM048298C21] Li, M. and Belmonte, J. C. I. (2017). Ground rules of the pluripotency gene regulatory network. *Nature reviews. Genetics* 18, 180-191. 10.1038/nrg.2016.156 28045100

[DMM048298C22] Liu, P., Cheng, H., Santiago, S., Raeder, M., Zhang, F., Isabella, A., Yang, J., Semaan, D. J., Chen, C., Fox, E. A. et al. (2011). Oncogenic PIK3CA-driven mammary tumors frequently recur via PI3K pathway-dependent and PI3K pathway-independent mechanisms. *Nat. Med.* 17, 1116-1120. 10.1038/nm.2402 21822287 PMC3169724

[DMM048298C23] Loh, Y. H., Wu, Q., Chew, J.-L., Vega, V. B., Zhang, W., Chen, X., Bourque, G., George, J., Leong, B., Liu, J. et al. (2006). The Oct4 and Nanog transcription network regulates pluripotency in mouse embryonic stem cells. *Nat. Genet.* 38, 431-440. 10.1038/ng1760 16518401

[DMM048298C24] Macleod, K. G., Serrels, B. and Carragher, N. O. (2017). Proteomics for drug discovery In *Methods in Molecular Biology* (ed. I. M. Lazar, M. Kontoyianni and A. C. Lazar), pp. 153-169. New York, NY: Springer New York.

[DMM048298C25] Madsen, R. R., Vanhaesebroeck, B. and Semple, R. K. (2018). Cancer-Associated PIK3CA Mutations in Overgrowth Disorders. *Trends Mol. Med.* 24, 856-870. 10.1016/j.molmed.2018.08.003 30197175 PMC6185869

[DMM048298C26] Madsen, R. R., Knox, R. G., Pearce, W., Lopez, S., Mahler-Araujo, B., Mcgranahan, N., Vanhaesebroeck, B. and Semple, R. K. (2019). Oncogenic PIK3CA promotes cellular stemness in an allele dose-dependent manner. *Proc. Natl Acad. Sci. USA* 116, 8380-8389. 10.1073/pnas.1821093116 30948643 PMC6486754

[DMM048298C27] Madsen, R. R., Rueda, O. M., Robin, X., Caldas, C., Semple, R. K. and Vanhaesebroeck, B. (2020). Relationship between stemness and transcriptionally-inferred PI3K activity in human breast cancer. *bioRxiv* 2020.07.09.195974. 10.1101/2020.07.09.195974

[DMM048298C28] Margaryan, N. V., Hazard-Jenkins, H., Salkeni, M., Smolkin, M., Coad, J., Wen, S., Seftor, E., Seftor, R. and Hendrix, M. (2019). The stem cell phenotype of aggressive breast cancer cells. *Cancers* 11, 1-11. 10.3390/cancers11030340 PMC6468512 30857267

[DMM048298C29] Mesnard, D., Guzman-Ayala, M. and Constam, D. B. (2006). Nodal specifies embryonic visceral endoderm and sustains pluripotent cells in the epiblast before overt axial patterning. *Development* 133, 2497-2505. 10.1242/dev.02413 16728477

[DMM048298C30] Mitsui, K. tokuzawa, Y., Itoh, H., Segawa, K., Murakami, M., Takahashi, K., Maruyama, M., Maeda, M. and Yamanaka, S. (2003). The homeoprotein nanog is required for maintenance of pluripotency in mouse epiblast and ES cells. *Cell* 113, 631-642. 10.1016/S0092-8674(03)00393-3 12787504

[DMM048298C31] Moniz, L. S. Surinova, S., Ghazaly, E., Velasco, L. G., Haider, S., Rodrã­Guez-Prados, J. C., Berenjeno, I. M., Chelala, C. and Vanhaesebroeck, B. (2017). Phosphoproteomic comparison of Pik3ca and Pten signalling identifies the nucleotidase NT5C as a novel AKT substrate. *Sci. Rep.* 7, 39985. 10.1038/srep39985 28059163 PMC5216349

[DMM048298C32] Murphy, D. J. junttila, M. R., Pouyet, L., Karnezis, A., Shchors, K., Bui, D. A., Brown-Swigart, L., Johnson, L. and Evan, G. I. (2008). Distinct thresholds govern Myc's biological output In Vivo. *Cancer Cell* 14, 447-457. 10.1016/j.ccr.2008.10.018 19061836 PMC2723751

[DMM048298C33] Nichols, J., Zevnik, B., Anastassiadis, K., Niwa, H., Klewe-Nebenius, D., Chambers, I., Schöler, H. and Smith, A. (1998). Formation of pluripotent stem cells in the mammalian embryo dependes on the POU transcription factor Oct4. *Cell* 95, 379-391. 10.1016/S0092-8674(00)81769-9 9814708

[DMM048298C34] Niwa, H., Miyazaki, J. and Smith, A. G. (2000). Quantitative expression of Oct-3/4 defines differentiation, dedifferentiation or self-renewal of ES cells. *Nat. Genet.* 24, 372-376. 10.1038/74199 10742100

[DMM048298C35] Parker, V. E. R., Keppler-Noreuil, K. M., Faivre, L., Luu, M., Oden, N. L., De Silva, L., Sapp, J. C., Andrews, K., Bardou, M., Chen, K. Y. et al. (2019). Safety and efficacy of low-dose sirolimus in the PIK3CA-related overgrowth spectrum. *Genet. Med.* 21, 1189-1198. 10.1038/s41436-018-0297-9 30270358 PMC6752269

[DMM048298C36] Pauklin, S. and Vallier, L. (2015). Activin/Nodal signalling in stem cells. *Development* 142, 607-619. 10.1242/dev.091769 25670788

[DMM048298C37] Perez-Riverol, Y., A. Csordas, J. Bai, M. Bernal-Llinares, S. Hewapathirana, D. J. Kundu, A. Inuganti, J. Griss, G. Mayer, M. Eisenacher et al. (2019). The PRIDE database and related tools and resources in 2019: Improving support for quantification data. *Nucleic Acids Res.* 47, D442-D450. 10.1093/nar/gky1106 30395289 PMC6323896

[DMM048298C38] Radzisheuskaya, A., Le Bin Chia, G., Dos Santos, R. L., Theunissen, T. W., Castro, L. F. C., Nichols, J. and Silva, J. C. R. (2013). A defined Oct4 level governs cell state transitions of pluripotency entry and differentiation into all embryonic lineages. *Nat. Cell Biol.* 15, 579-590. 10.1038/ncb2742 23629142 PMC3671976

[DMM048298C39] Robin, X., Voellmy, F., Ferkinghoff-Borg, J., Howard, C., Altenburg, T., Engel, M., Simpson, C. D., Saginc, G., Koplev, S., Klipp, E. et al. (2019). Probability-based detection of phosphoproteomic uncertainty reveals rare signaling events driven by oncogenic kinase gene fusion. *bioRxiv* 621961. 10.1101/621961

[DMM048298C40] Sanchez-Vega, F., Mina, M., Armenia, J., Chatila, W. K., Luna, A., La, K. C., Dimitriadoy, S., Liu, D. L., Kantheti, H. S., Saghafinia, S. et al. (2018). Oncogenic signaling pathways in the cancer genome atlas. *Cell* 173, 321-337.e10. 10.1016/j.cell.2018.03.035 29625050 PMC6070353

[DMM048298C41] Vallier, L., Reynolds, D. and Pedersen, R. A. (2004). Nodal inhibits differentiation of human embryonic stem cells along the neuroectodermal default pathway. *Dev. Biol.* 275, 403-421. 10.1016/j.ydbio.2004.08.031 15501227

[DMM048298C42] Vallier, L., Alexander, M. and Pedersen, R. A. (2005). Activin/Nodal and FGF pathways cooperate to maintain pluripotency of human embryonic stem cells. *J. Cell Sci.* 118, 4495-4509. 10.1242/jcs.02553 16179608

[DMM048298C43] Vallier, L., Mendjan, S., Brown, S., Chng, Z., Teo, A., Smithers, L. E., Trotter, M. W. B., Cho, C. H.-H., Martinez, A., Rugg-Gunn, P. et al. (2009). Activin/Nodal signalling maintains pluripotency by controlling Nanog expression. *Development*, 136, 1339-1349. 10.1242/dev.033951 19279133 PMC2687465

[DMM048298C44] Valvezan, A. J. and Manning, B. D. (2019). Molecular logic of mTORC1 signalling as a metabolic rheostat. *Nature Metabolism* 1, 321-333. 10.1038/s42255-019-0038-7 PMC12569966 32694720

[DMM048298C45] Vukicevic, S., Kleinman, H. K., Luyten, F. P., Roberts, A. B., Roche, N. S. and Reddi, A. H. (1992). Identification of multiple active growth factors in basement membrane Matrigel suggests caution in interpretation of cellular activity related to extracellular matrix components. *Exp. Cell Res.* 202, 1-8. 10.1016/0014-4827(92)90397-Q 1511725

[DMM048298C46] Xu, R. H., Sampsell-Barron, T. L., Gu, F., Root, S., Peck, R. M., Pan, G., Yu, J., Antosiewicz-Bourget, J., Tian, S., Stewart, R. et al. (2008). NANOG is a direct target of TGFβ/activin-mediated SMAD signaling in human ESCs. *Cell Stem Cell* 3, 196-206. 10.1016/j.stem.2008.07.001 18682241 PMC2758041

