# Supplementary material for: NODAL/TGFβ signalling mediates the self-sustained stemness induced by PIK3CAH1047R homozygosity in pluripotent stem cells
Source: Dis Model Mech. 2021 Mar 11;14(3):dmm048298. doi: 10.1242/dmm.048298 (PMC7969366; doi:10.1242/dmm.048298)
Supplement: Supplementary information [file dmm-14-048298-s1.pdf]

Supplementary Material Text

iPSC processing for individual experiments

Cell lysate collection for RNA sequencing and total proteomics

For RNA sequencing and total proteomics, subconfluent cells were fed fresh E8/F 3 h prior to snap-freezing on dry ice and subsequent RNA or protein extraction. Relative to the results in Ref. (Madsen *et al.*, 2019), the current transcriptomic data of *PIK3CA*^{H1047R} were obtained more than 6 months following the first study, with cells at different passages, and were thus independent from one another. Moreover, sample collection for the second transcriptomics experiment was conducted over three days according to a block design, thus allowing us to determine transcriptional differences that are robust to biological variability.

Cell lysate collection for RPPA

For RPPA in growth factor-replete conditions, cells were fed fresh E8/F 3h before collection. To assess variability due to differences in collection timing, clones from each iPSC genotype were collected on each one of three days according to a block design, giving rise to a total of 22 cultures. To test the effect of the PI3Kα-specific inhibitor BYL719, cells were treated with 100 nM drug (or DMSO only as control treatment) for 24 h and exposed to growth factor removal within the last hour before collection. All cells were washed in DPBS prior to collection to rinse off residual proteins and cell debris.

TGFβ/NODAL signalling studies

Wild-type or homozygous *PIK3CA*^{H1047R} iPSCs were seeded in 12-well plates all coated with Geltrex from the same lot (#2052962; diluted in DMEM/F12 lot #RNBH0692). Cells were processed for seeding at a ratio of 1:15 according to the standard maintenance protocol. One day after seeding, individual treatments were applied to triplicate wells. Briefly, cells were first washed twice with 2 and 1 ml of Dulbecco's PBS (DPBS) to remove residual growth factors. The base medium for individual treatments was Essential 6 supplemented with 10 ng ml^{-1} heat-stable FGF2. This was combined with one of the following reagents or their diluent equivalents: 100 ng ml^{-1} NODAL (diluent: 4 mM HCl), 250 nM BYL719 (diluent: DMSO), 5 μM SB431542 (diluent: DMSO). Cells were snap-frozen on dry ice after 24, 48 and 72 h following a single DPBS wash. Individual treatments were replenished daily at the same time of day to limit temporal confounders.

RNA sequencing data analyses

Raw read mapping, counting and differential expression

Raw reads were mapped to the human genome build GRCh38 (for iPSC RNAseq) or the mouse genome build GRCm38 (for MEF RNAseq), and gene level counts were performed using Spliced Transcripts Alignment to a Reference (STAR) v2.5 (Dobin *et al.*, 2013). Subsequent data processing was performed using open-source R software according to the *limma-voom* method (Ritchie *et al.*, 2015). Briefly, raw counts were converted to counts per million (cpm) using the *cpm()* function in the *edgeR* package (Robinson, McCarthy and Smyth, 2009), followed by normalisation according the trimmed mean of M (TMM) method (Robinson and Oshlack, 2010). The mean-variance relationship was modelled with *voom()*, followed by linear modelling and computation of moderated t-statistics using the *lmFit()* and *eBayes()* functions in the *limma* package (Ritchie *et al.*, 2015). The associated P-value for assessment of differential gene expression was adjusted for multiple comparisons with the Benjamini-Hochberg method at false-discovery rate (FDR) $\leq$ 5% (Benjamini and Hochberg, 1995). The function *duplicateCorrelation()* was applied to correct for the use of replicate iPSC clones.

Correlations between corresponding transcriptomics and/or proteomics data were calculated using Spearman's rank-order correlation test for non-normally distributed data.

Pathway and network analyses

Ingenuity® Pathway Analysis (IPA)

The list of differentially expressed total proteins (*PIK3CA*$^{H1047R/H1047R}$ *vs* wild-type) was subjected to IPA (build version: 448560M; content version: 36601845) against the Ingenuity Knowledge Base, considering only relationships where confidence was classified as "Experimentally Observed". Following exclusion of chemicals and drugs, the Upstream Regulators list was used for generation of Volcano plots of the respective activation z-scores and overlap p-values.

IPA was also used to analyse the lists of differentially expressed genes in both heterozygous and homozygous *PIK3CA*^{H1047R} iPSCs (IPA build version: 484108M; content version: 45868156) and MEFs (IPA build version: 486617M; content version: 46901286), using the Ingenuity Knowledge Base and considering only relationships where confidence was classified as "Experimentally Observed". Chemicals and diseases were excluded from Node Types. For the iPSC datasets, differentially expressed genes were only considered for IPA analysis if having an absolute log2(fold-change) $\geq$ log2(1.3). The choice of this relatively permissive log fold-change choice was guided by the *limma()* tutorial for RNAseq (Ritchie *et al.*, 2015), and the assumption that small fold-changes in the expression of genes that act within the same pathway may be sufficient to elicit a functionally important response and thus should not be omitted. This consideration is of particular relevance for transcriptomic data from heterozygous *PIK3CA*^{H1047R} iPSCs where fold-changes were relatively small. Due to the high number of differentially expressed transcripts in *PIK3CA*$^{H1047R/H1047R}$ iPSCs, the analysis was conducted using the top 2000 up- and top 2000 downregulated transcripts.

The IPA Upstream Regulator Analysis is based on the proprietary Ingenuity Knowledge Base which is used to compute two scores based on user-specified data: an enrichment score (Fisher's exact test p-value) that measures overlap between observed and predicted regulated gene sets; a z-score that assesses the match between observed and predicted up/down regulation patterns (Krämer *et al.*, 2014). The results of the Upstream Regulators Analysis were extracted for downstream Volcano plotting of overlap p-values and associated activation z-scores. Note that for heterozygous *PIK3CA*^{H1047R} iPSCs, a bias-corrected activation z-score was used for plotting to take into account any bias arising from a larger number of upregulated *vs* downregulated genes in these cells.

Weighted Gene Correlation Network Analysis (WGCNA)

RNA sequencing counts from all 12 samples were converted to reads per kilobase million (RPKM). A threshold of 10 RPKM was used to filter out low-expression genes, followed by removing any genes with missing values caused by this filtering. The RPKM values for the remaining 16,823 genes were log2 transformed and taken forward for network analysis using the *WGCNA* R package (Zhang and Horvath, 2005; Langfelder and Horvath, 2008), with a soft power threshold of 28 (chosen to maximise scale independence and minimise mean connectivity) and a minimum module size of 30 genes.

To identify modules associated with homozygosity for *PIK3CA*^{H1047R}, we used the correlation between a gene's module membership (eigengene) and significance for differential expression in homozygous *PIK3CA*$^{H1047R/H1047R}$ iPSCs. The top two most significant modules for the homozygosity trait were selected for functional enrichment analysis. CytoScape plugin ClueGO (version 2.5.4) (Bindea *et al.*, 2009) was used to perform pathway analysis using the Kyoto Encyclopedia of Genes and Genomes (KEGG) ontology (build 27.02.19) (Kanehisa *et al.*, 2017). All settings were kept at default values. Only pathways with p-value $\leq$ 0.05 were selected, and a custom reference gene set was used as background (the 16,823 genes analysed using WGCNA). Network visualisation was performed using Cytoscape (Shannon *et al.*, 2003).

Mass spectrometry data acquisition and analyses

Mass spectrometry (MS) data acquisition

All spectra were acquired on an Orbitrap Fusion Tribrid mass spectrometer (Thermo Fisher Scientific) operated in data-dependent mode coupled to an EASY-nLC 1200 liquid chromatography pump (Thermo Fisher Scientific) and separated on a 50 cm reversed phase column (Thermo Fisher Scientific, PepMap RSLC C18, 2 μM, 100A, 75 μm x 50 cm). Proteome samples (non-enriched) were eluted over a linear gradient ranging from 0-11% acetonitrile over 70 min, 11-20% acetonitrile for 80 min, 21-30% acetonitrile for 50 min, 31-48% acetonitrile for 30 min, followed by 76% acetonitrile for the final 10 min with a flow rate of 250 nl/min.

Survey-full scan MS spectra were acquired in the Orbitrap at a resolution of 120,000 from m/z 350-2000, automated gain control (AGC) target of 4x105 ions, and maximum injection time of 20 ms. Precursors were filtered based on charge state (≥2) and monoisotopic peak assignment, and dynamic exclusion was applied for 45s. A decision tree method allowed fragmentation for ion trap MS2 via electron transfer dissociation (ETD) or higher-energy collision dissociation (HCD), depending on charge state and m/z. Precursor ions were isolated with the quadrupole set to an isolation width of 1.6 m/z. MS2 spectra fragmented by ETD and HCD (35% collision energy) were acquired in the ion trap with an AGC target of 1e4. Maximum injection time for HCD and ETD was 80 ms for proteome samples.

Whole-exome sequencing (WES) and FASTA file generation

WES was performed on a single clone per genotype to generate cell-specific databases for downstream mass spectrometry searchers. Genomic DNA was extracted with Qiagen's QIAamp DNA Micro Kit according to the manufacturer's instructions, followed by quantification using the Qubit dsDNA High Sensitivity Assay Kit and by dilution to 5 ng/μl in the supplied TE buffer. The samples were submitted for library preparation and sequencing by the SMCL Next Generation Sequencing Hub (Academic Laboratory of Medical Genetics, Cambridge). Sequencing was performed on an Illumina HiSeq 4000 with 50X coverage across more than 60% of the exome in each sample. Raw reads were filtered with Trimmomatic (Bolger, Lohse and Usadel, 2014) using the following parameters: headcrop = 3, minlen = 30, trailing = 3. The trimmed reads were aligned to the human reference genome (hg19 build) with BWA (Li and Durbin, 2010), followed by application of GATK base quality score recalibration, indel realignment, duplicate removal and SNP/indel discovery with genotyping (McKenna *et al.*, 2010). GATK Best Practices standard hard filtering parameters were used throughout (Depristo *et al.*, 2011).

In order to find non-reference, mutated peptides in the MS data, we increased the search FASTA file with mutations affecting the protein sequence, as detected by WES with a high sensitivity filter: QD $\leq$ 1.5, FS $\geq$ 60, MQ $\geq$ 40, MQRankSum $\leq$ -12.5, ReadPosRankSum $\leq$ -8.0, and average DP $\geq$ 5 per sample. The Ensembl Variant Effect Predictor (VEP) with Ensembl v88 was used to predict the effect of the mutations on the protein sequence (McLaren *et al.*, 2016). For every variant with an effect on the protein sequence we added the predicted mutated tryptic peptide at the end of the protein sequence.

Mass spectrometry searches

Raw files were processed using MaxQuant 1.5.0.2 (Tyanova, Temu and Cox, 2016) with all searches conducted using cell-specific databases (see *Whole-exome sequencing and FASTA file generation*), where all protein sequence variants were included in addition to the reference (Ensemble v68 human FASTA). Methionine oxidation, protein N-terminal acetylation and serine/threonine/tyrosine phosphorylation were set as variable modifications and cysteine carbamidomethylation was set as a fixed modification. False discovery rates were set to 1% and the "match between runs" functionality was activated. We filtered out peptides that were associated with multiple identifications in the MaxQuant *msms.txt* file, had a score < 40, were identified in the reverse database or came from known contaminants. Analysis of the observed peptides passing these filters was performed using a Monte Carlo Markov Chain model as described previously (Robin *et al.*, 2019 preprint). Briefly, the model predicted the average ratio (sample versus control) of a peptide as a function of the observed protein concentration (obtained from the MaxQuant *evidence.txt* file). Combined with a noise model, a distribution of likely values for the parameters was obtained. The mean and standard deviation of this resulting distribution was used to calculate

a z-score which was used together with the fold-change (FC) for subsequent filtering for differentially expressed proteins ($|z| \geq 1.2$; $|\ln(FC)| \geq \ln(1.2)$).

RPPA data analyses

Slide images were analysed using Mapix software (Innopsys), with the spot diameter of the grid set to 270 µm. Background signal intensity was determined for each spot individually and subtracted from the sample spot signal. A test for linearity was performed from the four-point antibody dilution series, according to a flag system where $R^2 > 0.9$ was deemed good, $R^2 > 0.8$ was deemed acceptable and $R^2 < 0.8$ was poor (excluded from subsequent analyses). Median values from the four-point dilution series were calculated for each technical replicate and normalised to the corresponding Fast Green value to account for differences in protein loading. For each sample and protein target, a mean expression value was calculated from the remaining technical replicates and normalised to the corresponding mean of the wild-type group. All phosphoprotein signals were also normalised to the corresponding total protein values.

RT-qPCR set-up and data analyses

For SYBR Green-based qPCRs, A 5-fold cDNA dilution series was prepared and used as standard curve for relative quantitation of gene expression. *TBP* was used as normaliser following confirmation that its gene expression remained unaffected by the tested conditions. Melt curve analyses confirmed amplification of a single product by each primer pair. All primers had amplification efficiencies 95%-105%. Samples were loaded in duplicate in 384-well plates.

The TaqMan hPSC Scorecard was set up according to the manufacturer's instructions with the following modifications. From each cDNA sample diluted to 20 ng/µl, two 50 µl RT reactions were set up, with 500 ng RNA sample in each. Next, the two RT replicates were combined to obtain 1 µg cDNA in a total volume of 100 µl (final concentration: 10 ng/µl). This was subsequently diluted to 0.715 ng/µl and 10 µl loaded into each Scorecard well. All Ct values were mapped to their corresponding genes using the TaqMan hPSC Scorecard analysis software provided by the manufacturer. Genes with Ct values < 15 were excluded from further analyses. To be considered for downstream processing, genes were also required to have Ct values < 30 in at least two out of the eight samples. Next, Ct values were linearised (antilog) under the assumption of 100 % primer amplification efficiency. The geometric expression mean of the control gene assays was used for subsequent normalisation of individual gene expression values.

All qPCR data were acquired on a Quant Studio™ 5 Real-Time PCR System (Thermo Fisher Scientific). The thermocycling conditions (SYBR Green reactions) were as follows (ramp rate 1.6°C/s for all): 50°C for 2 min, 95°C for 10 min, 40 cycles at 95°C for 15 sec and 60°C for 1 min, followed by melt curve analysis (95°C for 15 sec, 60°C for 1 min, and 95°C for 15 min with ramp rate 0.075°C/sec). The TaqMan hPSC Scorecard thermocycling conditions were as specified by the manufacturer in the accompanying template.

All relevant primer sequences are included in **Table S4**.

Colony size quantitation from light micrographs

Routine cell culture light micrographs were acquired on an EVOS FL digital inverted microscope (AMF4300, Thermo Fisher Scientific) using the 4X or 10X objective (final magnification 40X and 100X, respectively). For quantitation, 4X images were used for colony segmentation with Definiens Developer XD software. Background was detected using a contrast threshold; for this each pixel was compared to those in the surrounding 24 pixels (i.e. a 5x5 pixel box), and pixels with low contrast (between -50 and +50) were classified as background. Remaining pixels were classified as colonies, and any holes (pixels that were not initially classified as being part of the colony due to low contrast) were filled. Edges of the resulting colonies were smoothened by shrinking and then growing the colonies by 2 pixels. Finally, colonies less than 2000 pixels in size were reclassified as background. The area of the resulting colonies could then be measured and averaged over each field of view.

Disease Models & Mechanisms • Supplementary information

Statistical analyses

In line with recent (ATOMIC) recommendations by the American Statistical Association (Wasserstein, Schirm and Lazar, 2019), we have avoided arbitrary use of “statistical significance” applied to data from small-scale cell culture experiments which violate assumptions of the most widely used statistical tests. Instead, we present all data from multiple orthogonal experiments, alongside complete information on experimental replicates, independent clones and replicate cultures.

For RPPA data in Fig. 2A, a statistical test for differential expression was performed on datasets with more than three samples per group, using the *limma* package to apply the *limma-trend* method with *lmFit()* and *eBayes()*, specifying collection time as blocking factor (Ritchie *et al.*, 2015). Phosphoprotein and total protein lists were processed separately. The associated p-value for assessment of differential gene expression was adjusted for multiple comparisons with the Benjamini-Hochberg method at FDR $\leq$ 5% (Benjamini and Hochberg, 1995). The function *duplicateCorrelation()* was applied to correct for the use of replicate iPSC clones on the same day. Heatmaps were generated using the *heatmap.2()* function within the *gplots* package in R, using target-wise correlation for dendrogram construction.

Disease Models & Mechanisms • Supplementary information

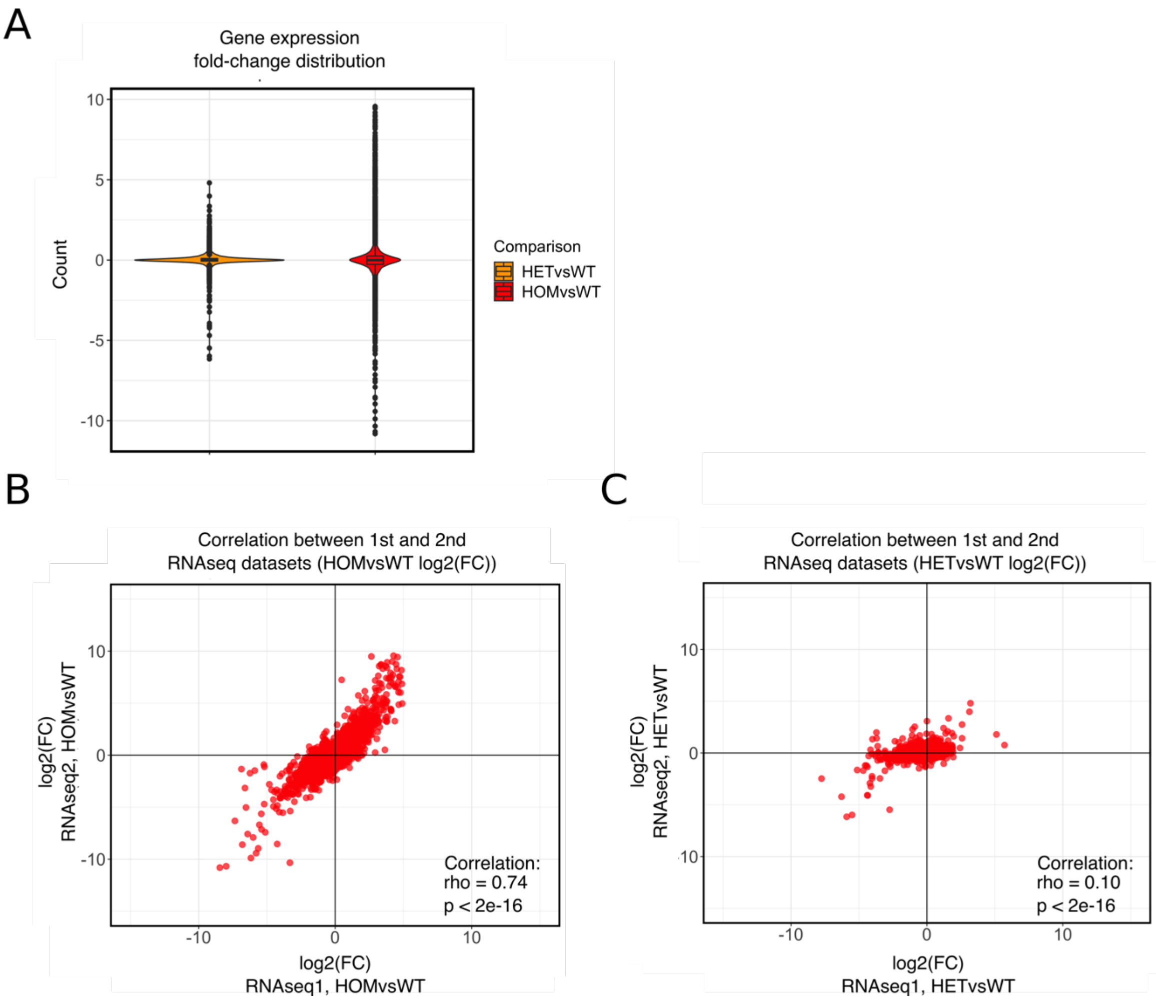

Fig. S1, related to Fig. 1. Fold-change distribution and transcriptome correlations. (A) Combined violin-boxplot representation of the log2 expression fold-change (FC) distribution of gene expression changes between heterozygous and homozygous *PIK3CA*^{H1047R} and wild-type iPSCs as indicated. (B) and (C) Correlation plot of the log2 expression fold-changes (FC) of significant total proteins vs the corresponding mRNA transcripts in *PIK3CA*$^{H1047R/H1047R}$ (HOM) (B) and *PIK3CA*$^{WT/H1047R}$ (HET)(C) iPSCs. Spearman's rho and the corresponding P-values are indicated on all plots. The first transcriptomic study (RNAseq1) was based on three independent cultures from three different clones per genotype; the second transcriptomic study (RNAseq2) used four independent cultures per genotype, from at least two independent clones each.

Disease Models & Mechanisms • Supplementary information

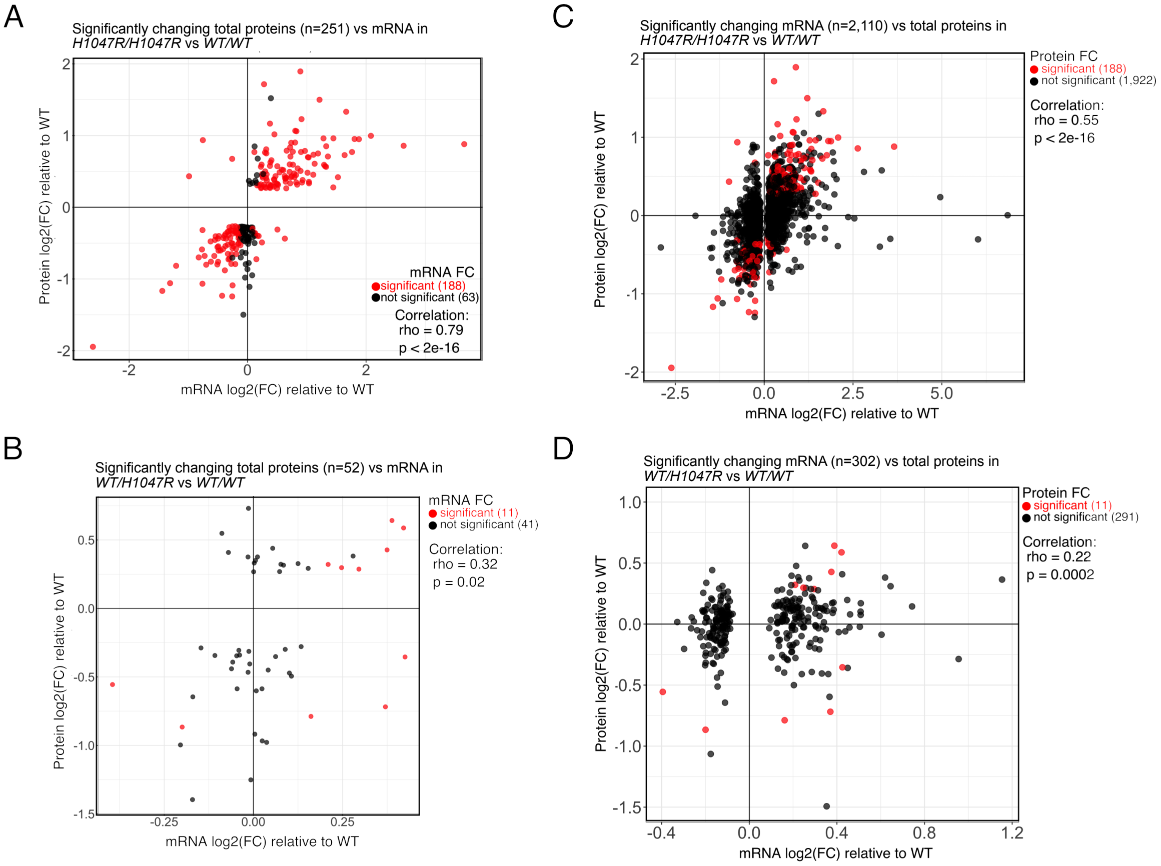

Fig. S2, **related to Fig. 1. Transcriptome-proteome correlations.** (A) Correlation plot of the log2 expression fold-changes (FC) of differentially expressed total proteins (|z| ≥ 1.2 and |ln(FC)| ≥ 1.2) in *PIK3CA*$^{H1047R/H1047R}$ iPSCs and the corresponding mRNA transcripts in an independent set of cultures. If identified as differentially expressed following statistical analysis (FDR ≤ 0.05), mRNA transcripts are highlighted in red irrespective of absolute fold-change. (B) As in (A) but starting with all differentially expressed mRNA transcripts (FDR ≤ 0.05 irrespective of fold-change magnitude) and plotting them to the corresponding protein identified by total proteomics. If differentially expressed (see (a)), the matched proteins are highlighted in red. (C) and (D) As in (A) and (B), respectively, but using the data for *PIK3CA*$^{WT/H1047R}$ iPSCs. All proteomic data were obtained from 3 independent clones per genotype using cultures at passages P47-P52, corresponding to the cultures used in our previous study (Madsen *et al.*, 2019). The high-depth transcriptomic data were obtained from 4 independent cultures (minimum 2 independent clones) per genotype using cultures at passages P55-P59. Spearman's rho and the corresponding P-values are indicated on all plots.

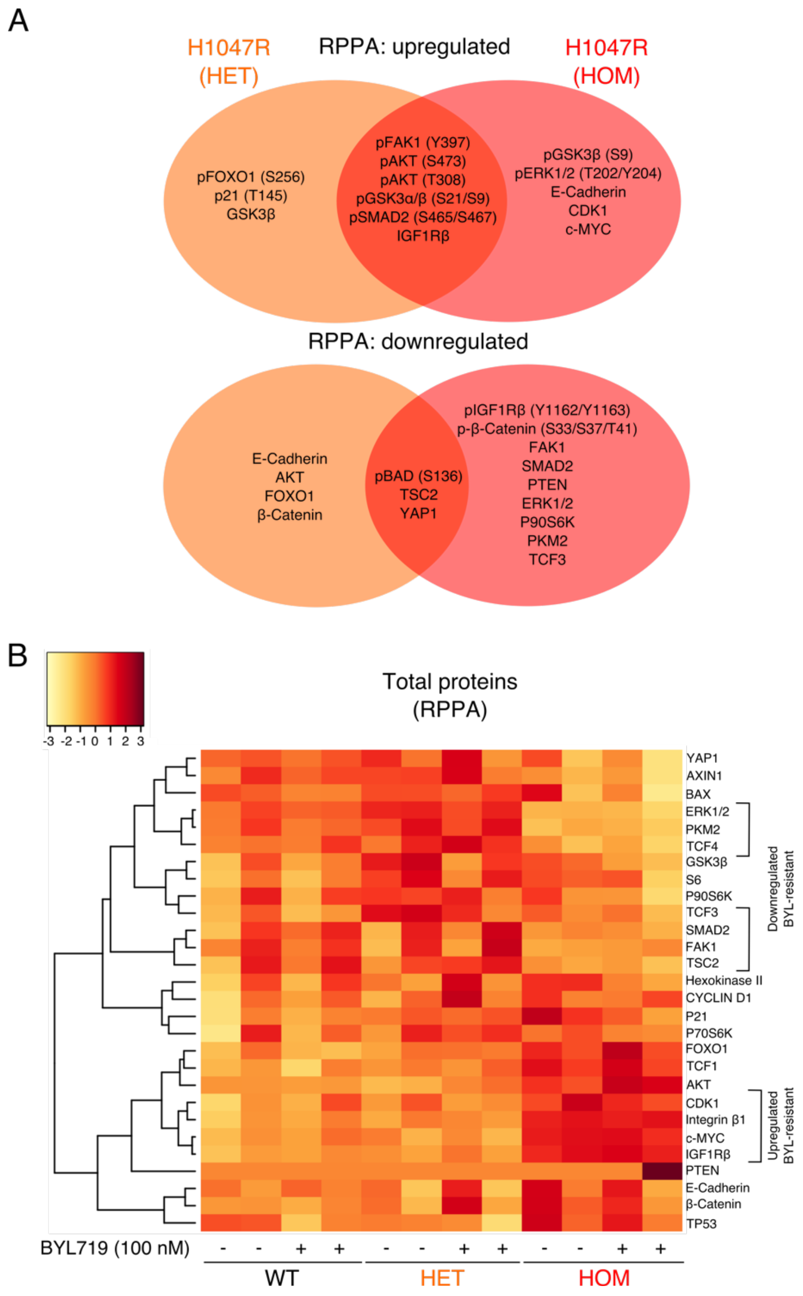

Fig. S3, related to Fig. 2. Additional RPPA data. (A) Venn-diagrams specifying differentially expressed phosphorylated and total proteins in *PIK3CA*$^{WT/H1047R}$ (HET) and *PIK3CA*$^{H1047R/H1047R}$ (HOM) iPSCs relative to wild-type controls, based on RPPA profiling of cells cultured in growth factor-replete conditions. The data are based on a total of 10 wild-type cultures, 5 *PIK3CA*$^{WT/H1047R}$ cultures and 7 *PIK3CA*$^{H1047R/H1047R}$ cultures, and all shown targets were differentially expressed at a false-discovery rate (FDR) $\leq$ 0.05. Following quality checks (see Materials and Methods), the RPPA data included 21 phosphorylated and 21 total proteins. (B) Heatmap of total proteins from RPPA profiling of wild-type (WT), *PIK3CA*$^{WT/H1047R}$ (HET) and *PIK3CA*$^{H1047R/H1047R}$ (HOM) iPSCs following short-term growth factor removal (1 h), +/- 100 nM BYL719 (PI3Kα inhibitor) for 24 h. Groups of total proteins exhibiting a consistent expression pattern in BYL719-treated *PIK3CA*$^{H1047R/H1047R}$ iPSCs are specified.

Disease Models & Mechanisms • Supplementary information

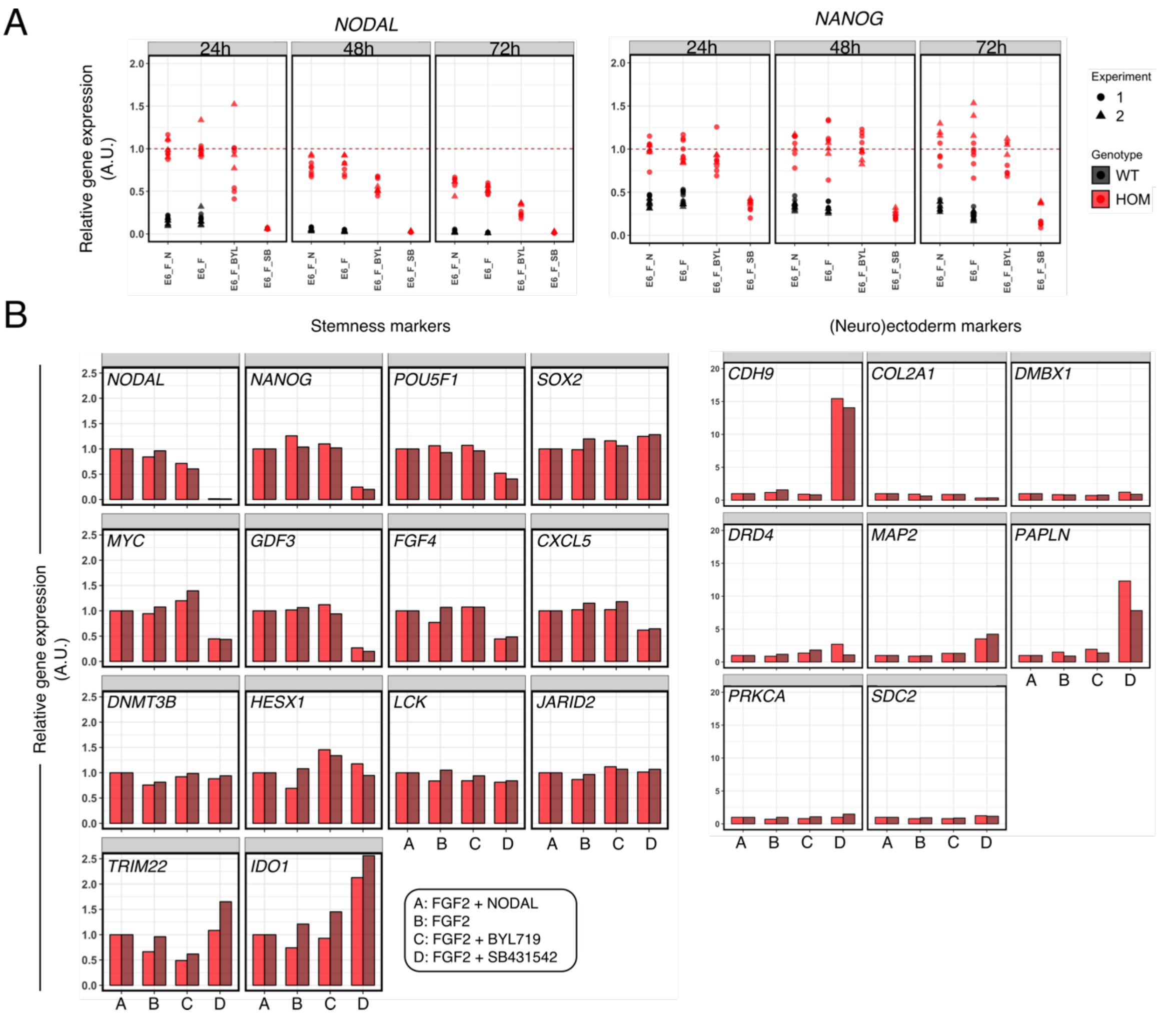

Fig. S4, **related to Fig. 5. Alternative representation of the experimental data in Fig. 5A and additional RT-qPCR-based profiling of lineage-specific markers.** (A) As in Fig. 5A but representing individual gene expression values for *NODAL* and *NANOG* scaled to the mean expression in $PIK3CA^{H1047R/H1047R}$ after 24 h in E6 medium supplemented with FGF2 and NODAL. A.U., arbitrary units. (B) TaqMan hPSC Scorecards were used to profile a set of stemness and (neuro)ectoderm markers in $PIK3CA^{H1047R/H1047R}$ iPSCs following the indicated treatments for 48 h. Note that *FGF4, GDF3, MYC* and *JARID2* were manually classified as stemness markers based on previous large scale characterisations of human pluripotent stem cells (Adewumi *et al.*, 2007). Each bar corresponds to a single sample, with colours specifying the use of two independent clones per treatment. For each gene and clone, values are scaled to the expression value in cells cultured in E6 medium with FGF2 and NODAL.

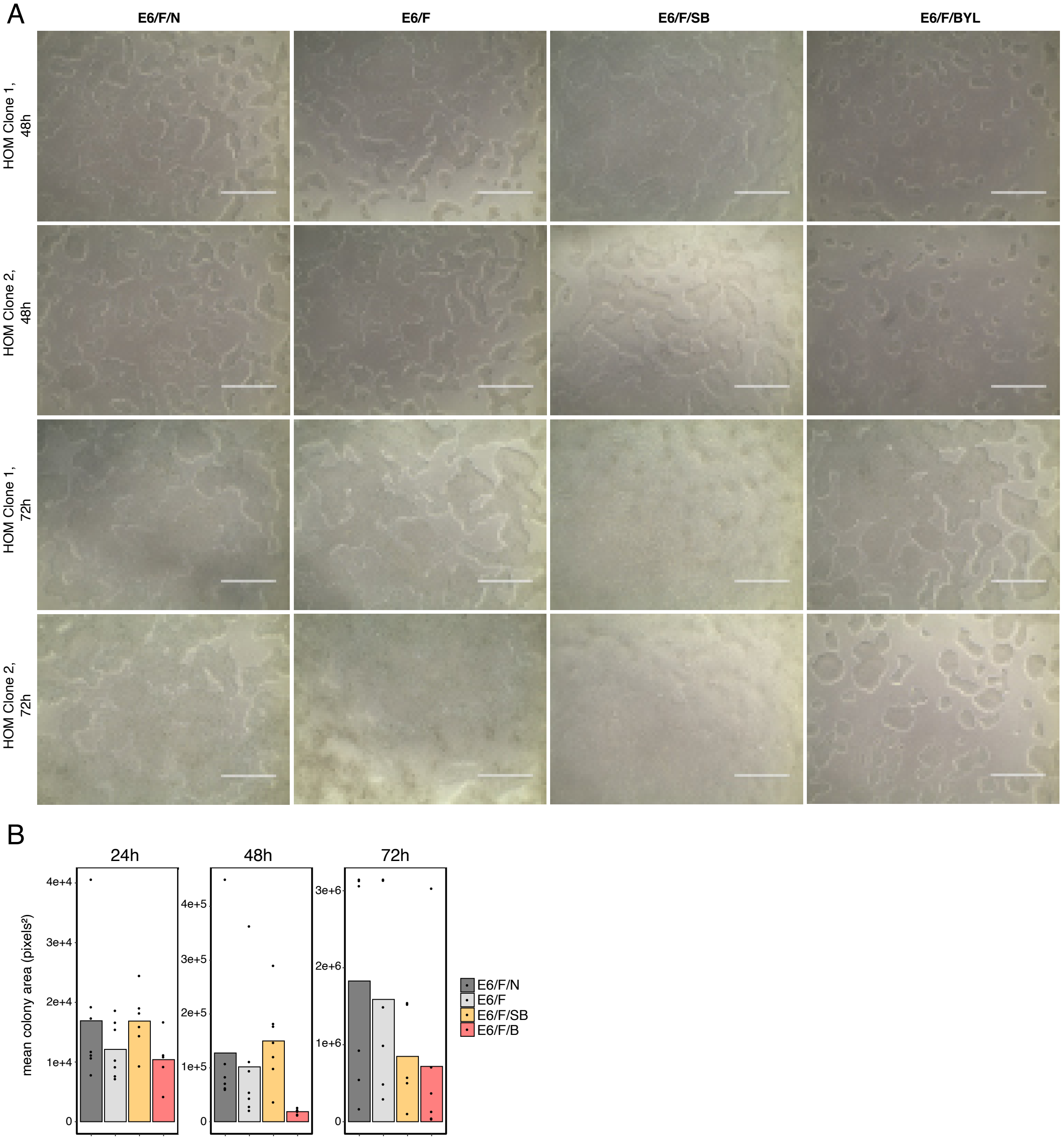

Fig. S5, related to Fig. 5. Light micrographs of *PIK3CA*$^{H1047R/H1047R}$ iPSC clones exposed to different media formulations +/- specific inhibitors. A. Each row corresponds to images of parallel cultures of one of two independent iPSC clones exposed to different treatments for 48 or 72 h as indicated. E6/F/N: Essential 6 medium supplemented with 10 ng ml^{-1} FGF2 and 100 ng ml^{-1} NODAL; E6/F: Essential 6 medium supplemented with 10 ng ml^{-1} FGF2; E6/F/SB: Essential 6 medium supplemented with 10 ng ml^{-1} FGF2 and 5 μM SB431542; E6/F/BYL: Essential 6 medium supplemented with 10 ng ml^{-1} FGF2 and 250 nM BYL719. The images are representative of 3 independent experiments. Scale bar: 1000 μm. The original images at higher resolution are available online on https://osf.io/hbf7x/. **B.** Quantification of colony size based on the acquired light micrographs; individual points corresponding to separate images and bars represent the overall mean across the three experimental replicates.

Cell line	Source	Identifier
WTC11 male parental iPSC line	Coriell Institute	GM25256
WTC11-derived male wild-type post-CRISPR editing	Madsen *et al.* PNAS 2019 (Madsen *et al.*, 2019)	N/A
WTC11-derived male *PIK3CA*$^{WT/H1047R}$	Madsen *et al.* PNAS 2019 (Madsen *et al.*, 2019)	N/A
WTC11-derived male *PIK3CA*$^{H1047R/H1047R}$	Madsen *et al.* PNAS 2019 (Madsen *et al.*, 2019)	N/A
M98-WT female iPSC	Madsen *et al.* PNAS 2019 (Madsen *et al.*, 2019)	N/A
M98-E418K (*PIK3CA*$^{WT/E418K}$) female iPSC	Madsen *et al.* PNAS 2019 (Madsen *et al.*, 2019)	N/A
Primary MEFs *PIK3CA*$^{WT/WT}$ (H1047R uninduced)	Moniz *et al.* Sci Reports 2017 (Moniz *et al.*, 2017)	N/A
Primary MEFs *PIK3CA*$^{WT/H1047R}$ (induced for 48h)	Moniz *et al.* Sci Reports 2017 (Moniz *et al.*, 2017)	N/A

Table S1. Cell lines.

Disease Models & Mechanisms • Supplementary information

Item	Catalogue no.	Vendor	Application
100 ml DMSO, sterile-filtered	D2650	Sigma Aldrich	Cells
2X PowerUp SYBR Green Master Mix	A25742	Thermo Fisher Scientific	Molbio
Acetonitrile	1.00029	Sigma Aldrich	Protein work
Amonium Bicarbonate	09830	Sigma Aldrich	Protein work
Bioanalyzer RNA 6000 Nano Kit	5067-1511	Agilent	Molbio
BYL719 (lot #2598155)	B9700-1mg	Cambridge Bioscience	Cells
Chloroacetamide	C0267	Sigma Aldrich	Protein work
cOmplete ULTRA Tablets, EDTA-free	5892791001	Roche	Protein work
cOmplete ULTRA Tablets, Mini, EasyPack, PhosStop	4906845001	Roche	Protein work
DC Protein Assay Reagent A	5000113	Biorad	Protein
DC Protein Assay Reagent B	5000114	Biorad	Protein
DC Protein Assay Reagent S	5000115	Biorad	Protein
DirectZol RNA Miniprep Kit	R2051	ZymoResearch	Molbio
Dithiothritol	10197777001	Sigma Aldrich	Protein work
DMEM/F12 without L-Glutamine, HEPES 500 ml	D6421	Sigma Aldrich	Cells
DPBS without Ca^{2+} and Mg^{2+}	D8537	Sigma Aldich	Cells
EndoFree Plasmid Maxi Kit (10)	12362	Qiagen	Molbio
Endopeptidase Lys-C	129-02541	Wako	Protein work
Essential 6 Medium	A1516401	Thermo Fisher Scientific	Cells
Essential 8 Flex Medium Kit	A2858501	Thermo Fisher Scientific	Cells
Geltrex LDEV Free hESC Quality 5 ml	A1413302	Thermo Fisher Scientific	Cells
Gibco Heat Stable Recombinant Human bFGF (for prolonged culture), 100 ug (lot #2046190)	PHG0369	Thermo Fisher Scientific	Cells
HEPES	H4034	Sigma Aldrich	Protein work
High-Capacity cDNA Reverse Transcription Kit	4368814	Thermo Fisher Scientific	Molbio
InSolution™ TGF-b RI Kinase Inhibitor VI, SB431542 (lot #3075261)	616464-5MG	Merck Millipore	Cells
Nuclease-free H_2O	AM9932	Thermo Fisher Scientific	Molbio/Portein work
Nunc™ Delta Cell-Culture Treated Multidishes; 6-well	140685	Thermo Fisher Scientific	Cells
Nunc™ Delta Cell-Culture Treated Multidishes; 12-well	150628	Thermo Fisher Scientific	Cells
Pierce Quantitative Colorimetric Peptide Assay	23275	Thermo Fisher Scientific	Protein work

Disease Models & Mechanisms • Supplementary information

QIAamp DNA Micro Kit (50)	56304	Qiagen	Molbio
QIAshredder	79654	Qiagen	Molbio
QIAzol Lysis Reagent	79306	Qiagen	Molbio
Quibit dsDNA HS Assay Kit	Q32854	Thermo Fisher Scientific	Molbio
Recombinant human NODAL protein, 25 ug (lot #OLF1115091)	3218-ND/CF	R&D	Cells
ReLeSR 100 ml	5872	Stem Cell Technologies	Cells
RevitaCell Suppplement 5 ml	A2644501	Thermo Fisher Scientific	Cells
RNeasy Mini Kit	74104	Qiagen	Molbio
SepPak C18 cartridges	WAT02051 5	Waters	Protein work
SuperScript IV Fist Strand Synthesis System	18091050	Thermo Fisher Scientific	Molbio
Urea	15604	Sigma Aldrich	Protein work
TaqMan® hPSC Scorecard™ Panel, 384-well	A15870	Thermo Fisher Scientific	Molbio
Thiourea	T7875	Sigma Aldrich	Protein work
Trifluoroacetic acid	T6508	Sigma Aldrich	Protein work
TrueSeq Stranded mRNA Library Prep Kit	20020594	Illumina	Molbio
TruSeq RNA Library Prep Kit v2	RS-930-2002	Illumina	Molbio
TruSeq RNA Single Indexes Set A	20020492	Illumina	Molbio
Trypsin	T6567	Sigma	Protein work
VenorGeM Classic Mycoplasma Kit	11-1005	Minerva Biolabs	Molbio

Table S2, Materials list. Molbio, molecular biology.

Disease Models & Mechanisms • Supplementary information

Primary antibody (clone if mAb)	Vendor	Catalogue #	Lot #	Species
4E-BP1	CST	9452	10	rabbit
4E-BP1 P Ser65	CST	9451	14	rabbit
4E-BP1 P Ser65 (174A9)	CST	9456	5	rabbit
4E-BP1 P Thr37,Thr46	CST	9459	8	rabbit
Acetyl-CoA carboxylase P Ser79	CST	3661	10	rabbit
AKT	CST	9272	24	rabbit
AKT P Ser473	CST	9271	13	rabbit
AKT P Thr308	CST	9275	19	rabbit
AXIN1 (C76H11)	CST	2087	4	rabbit
β -Catenin	CST	9562	11	rabbit
β -Catenin P Ser33,Ser37,Thr41	CST	9561	12	rabbit
β -Catenin P Thr41,Ser45	CST	9565	2	rabbit
BAD	CST	9292		rabbit
BAD P Ser112	CST	9291	8	rabbit
BAD P Ser136	CST	9295	11	rabbit
BAX	CST	2772	8	rabbit
c-MYC	CST	5605	8	rabbit
c-MYC P Thr58,Ser62	Epitomics	1203-1		rabbit
CDK1	CST	9112	5	rabbit
CDK1 P Tyr15	CST	9111	12	rabbit
Cyclin D1	CST	2926	10	mouse
Cyclin D1 P Thr286	CST	3300	3	rabbit
E-Cadherin	CST	3195	13	rabbit
ERK1/2	CST	9102	26	rabbit
ERK1/2 P CST Thr202/Thr185,Tyr204/Tyr187	CST	4370	12	rabbit
FAK1	CST	3285	9	rabbit
FAK1 P Y397	CST	3283	2	rabbit
FOXO1 P S256	CST	9461	7	rabbit
FOXO1 (C29H4)	CST	2880	10	rabbit
GSK3α/β P Ser21/Ser9	CST	9331	15	rabbit
GSK3β	CST	9315	12	rabbit
GSK3β P Ser9	CST	9336	12	rabbit
Hexokinase II	CST	2867	3	rabbit
IGF1Rβ	CST	3027	13	rabbit
IGF1Rβ P Tyr1162,Tyr1163	Invitrogen (Biosource)	44-804G	633298A	rabbit
Integrin β 1 (EP1041Y)	abcam	ab52971		rabbit
Lamin A/C	CST	2032	5	rabbit
LEF1 (C12A5)	CST	2230	5	rabbit
P21	CST	2946	14	mouse
P21 p Thr145	Santa Cruz	sc-20220-R	A2811	rabbit
p110α	CST	4249	7	rabbit
PKM2 XP(R)	CST	4053	3	rabbit
PKM2 P Tyr105	CST	3827	3	rabbit
PTEN	CST	9552	3	rabbit
RB P Ser780	CST	9307	13	rabbit
Rictor P T1135	CST	3806		rabbit
RPS6KA (Rsk1-3)	Santa Cruz	sc-231	I0110	rabbit
RPS6KA (Rsk1-3) P Thr359,Ser363	CST	9344	8	rabbit
S6	CST	2217	5	rabbit

S6 P Ser235,Ser236	CST	2211	20	rabbit
S6 P Ser240,Ser244	CST	2215	14	rabbit
S6K1	CST	9202	15	rabbit
S6K1 P Thr389	CST	9205S	16	rabbit
SLUG (C19G7)	CST	9585	5	rabbit
SMAD2 (C86F7)	CST	3122	8	rabbit
SMAD2 P Ser465,Ser467	CST	3108	7	rabbit
SMAD2/3 P Ser465/Ser423, Ser467/Ser425	CST	8828	6	rabbit
SMAD3 P Ser423,Ser425	CST	9520	13	rabbit
TCF1 (C63O9)	CST	2203	6	rabbit
TCF3 (D15G11)	CST	2883	4	rabbit
TCF4 (C9B9)	CST	2565	3	rabbit
TGFβ (56E4)	CST	3709	4	rabbit
TP53	CST	9282	4	rabbit
TSC2	CST	3612	3	rabbit
TSC2 P Thr1462	CST	3617	4	rabbit
YAP P Ser127	CST	4911	2	rabbit
YAP1 (EP1674Y)	Abcam	ab52771		rabbit
**Secondary antibody**				
Anti Rabbit Dylight 800 conjugated	CST	5151		goat
Anti Mouse Dylight 800 conjugated	CST	5257		goat

Table S3. Primary and secondary antibodies used for RPPA. All primary antibodies were diluted 1:250 in Superblock T20 (Thermo Fisher Scientific #37536). Secondary antibodies were also diluted in Superblock, at 1:2500. CST, Cell Signaling Technology. mAb, monoclonal antibody.

Disease Models & Mechanisms • Supplementary information

Target	Accession ID (RefSeq)	Fwd primer	Rev primer	Amplicon size (bp)
NANOG	NM_024865.3	CAGTCTGGACACTGGCTGAA	CTCGCTGATTAGGCTCCAAC	149
NODAL	NM_018055.4	CAGTACAACGCCTATCGCTGT	TGCATGGTTGGTCGGATGAAA	75
POU5F1 (OCT3/4)	NM_002701.5	TGTACTCCTCGGTCCCTTTC	TCCAGGTTTTCTTTCCCTAGC	150
TBP	NM_003194.4	TAATCCCAAGCGGTTTGC	TAGCTGGAAAACCCAACTTCT	170

Table S4. SYBR Green qPCR primers. Bp, base pairs; Fwd, forward; Rev; reverse. Only a single accession ID is given due to space constraints, but all primers detect additional splice isoforms of their specific target.

Disease Models & Mechanisms • Supplementary information

Table S5. List of differentially expressed genes in *PIK3CA*$^{WT/H1047R}$ vs wild-type hPSCs after applying an absolute fold-change cut-off of minimum 1.3.

Click here to Download Data S1

Table S6. List of differentially expressed genes in *PIK3CA*$^{H1047R/H1047R}$ vs wild-type hPSCs after applying an absolute fold-change cut-off of minimum 1.3.

Click here to Download Data S2

Table S7. List of differentially expressed genes in *PIK3CA*$^{WT/E418K}$ vs wild-type hPSCs.

Click here to Download Data S3

Table S8. List of differentially expressed genes in *PIK3CA*$^{WT/H1047R}$ vs wild-type MEFs

Click here to Download Data S4

Table S9. List of differentially expressed proteins in *PIK3CA*$^{WT/H1047R}$ vs wild-type hPSCs.

Click here to Download Data S5

Table S10. List of differentially expressed proteins in *PIK3CA*$^{H1047R/H1047R}$ vs wild-type hPSCs.

Click here to Download Data S6

Table S11. List of differentially expressed proteins changing in the same direction in both heterozygous and homozygous *PIK3CA*^{H1047R} hPSCs vs wild-type controls.

Click here to Download Data S7

Table S12. List of differentially expressed transcripts changing in the same direction in both heterozygous and homozygous *PIK3CA*^{H1047R} hPSCs vs wild-type controls.

Click here to Download Data S8

Source data deposited on the Open Science Framework

- High_depth_iPSC_H1047R_RNAseq (related to Fig. 1, 3; Fig. S1, S2)
- M98_iPSCs_E418K_RNaseq (related to Fig. 1)
- MEF_H1047R_RNAseq (related to Fig. 1)
- Total_proteomics (related to Fig. 1, 3; Fig. S2)
- GF_replete_iPSCs_H1047R_RPPA_study (related to Fig. 2; Fig. S3)
- BYL719_iPSCs_H1047R_RPPA_study (related to Fig. 2; Fig. S3)
- WGCNA_iPSC_RNAseq_analysis (related to Fig. 4)
- NODAL_experiment (related to Fig. 5; Fig. S4)

Disease Models & Mechanisms • Supplementary information

References

Adewumi, O. *et al.* (2007) 'Characterization of human embryonic stem cell lines by the International Stem Cell Initiative', *Nature Biotechnology*, 25(7), pp. 803–816. doi: 10.1038/nbt1318.

Benjamini, Y. and Hochberg, Y. (1995) 'Controlling the False Discovery Rate - a Practical and Powerful Approach to Multiple Testing', *Journal of the Royal Statistical Society Series B-Methodological*, 57, pp. 289–300.

Bindea, G. *et al.* (2009) 'ClueGO: a Cytoscape plug-in to decipher functionally grouped gene ontology and pathway annotation networks', *Bioinformatics*, 25(8), pp. 1091–1093. doi: 10.1093/bioinformatics/btp101.

Bolger, A. M., Lohse, M. and Usadel, B. (2014) 'Trimmomatic: A flexible trimmer for Illumina sequence data', *Bioinformatics*. doi: 10.1093/bioinformatics/btu170.

Depristo, M. A. *et al.* (2011) 'A framework for variation discovery and genotyping using next-generation DNA sequencing data', *Nature Genetics*, 43(5), pp. 491–501. doi: 10.1038/ng.806.

Dobin, A. *et al.* (2013) 'STAR: Ultrafast universal RNA-seq aligner', *Bioinformatics (Oxford, England)*, 29(1), pp. 15–21. doi: 10.1093/bioinformatics/bts635.

Kanehisa, M. *et al.* (2017) 'KEGG: New perspectives on genomes, pathways, diseases and drugs', *Nucleic Acids Research*, 45(D1), pp. D353–D361. doi: 10.1093/nar/gkw1092.

Krämer, A. *et al.* (2014) 'Causal analysis approaches in ingenuity pathway analysis', *Bioinformatics*, 30(4), pp. 523–530. doi: 10.1093/bioinformatics/btt703.

Langfelder, P. and Horvath, S. (2008) 'WGCNA: an R package for weighted correlation network analysis.', *BMC bioinformatics*, 9, p. 559. doi: 10.1186/1471-2105-9-559.

Li, H. and Durbin, R. (2010) 'Fast and accurate long-read alignment with Burrows–Wheeler transform', *Bioinformatics*, 26(5), pp. 589–595. doi: 10.1093/bioinformatics/btp698.

Madsen, R. R. *et al.* (2019) 'Oncogenic PIK3CA promotes cellular stemness in an allele dose-dependent manner', *Proceedings of the National Academy of Sciences*, 116(17), pp. 8380–8389. doi: 10.1073/pnas.1821093116.

McKenna, A. *et al.* (2010) 'The Genome Analysis Toolkit: A MapReduce framework for analyzing next-generation DNA sequencing data', *Genome Research*, 20(9), pp. 1297–1303. doi: 10.1101/gr.107524.110.

McLaren, W. *et al.* (2016) 'The Ensembl Variant Effect Predictor', *Genome Biology*, 17(1), p. 122. doi: 10.1186/s13059-016-0974-4.

Moniz, L. S. *et al.* (2017) 'Phosphoproteomic comparison of Pik3ca and Pten signalling identifies the nucleotidase NT5C as a novel AKT substrate', *Scientific Reports*, 7, p. 39985. doi: 10.1038/srep39985.

Ritchie, M. E. *et al.* (2015) 'limma powers differential expression analyses for RNA-sequencing and microarray studies', *Nucleic Acids Research*, 43(7), pp. e47–e47. doi: 10.1093/nar/gkv007.

Robin, X. *et al.* (2019) 'Probability-based detection of phosphoproteomic uncertainty reveals rare signaling events driven by oncogenic kinase gene fusion', *bioRxiv*, p. 621961. doi:

10.1101/621961.

Robinson, M. D., McCarthy, D. J. and Smyth, G. K. (2009) 'edgeR: A Bioconductor package for differential expression analysis of digital gene expression data', *Bioinformatics*. doi: 10.1093/bioinformatics/btp616.

Robinson, M. D. and Oshlack, A. (2010) 'A scaling normalization method for differential expression analysis of RNA-seq data', *Genome Biology*, 11(3), p. R25. doi: 10.1186/gb-2010-11-3-r25.

Shannon, P. *et al.* (2003) 'Cytoscape: a software environment for integrated models of biomolecular interaction networks.', *Genome research*, 13(11), pp. 2498–504. doi: 10.1101/gr.1239303.

Tyanova, S., Temu, T. and Cox, J. (2016) 'The MaxQuant computational platform for mass spectrometry-based shotgun proteomics.', *Nature protocols*. Nature Publishing Group, 11(12), pp. 2301–2319. doi: 10.1038/nprot.2016.136.

Wasserstein, R. L., Schirm, A. L. and Lazar, N. A. (2019) 'Moving to a World Beyond " p < 0.05"', *The American Statistician*. Taylor & Francis, 73(sup1), pp. 1–19. doi: 10.1080/00031305.2019.1583913.

Zhang, B. and Horvath, S. (2005) 'A General Framework for Weighted Gene Co-Expression Network Analysis', *Statistical Applications in Genetics and Molecular Biology*, 4(1). doi: 10.2202/1544-6115.1128.

Disease Models & Mechanisms • Supplementary information
